# Search for dark matter at $\sqrt{s}=13~\mathrm{TeV}$ in final states containing an energetic photon and large missing transverse momentum with the ATLAS detector

**DOI:** 10.1140/epjc/s10052-017-4965-8

**Published:** 2017-06-14

**Authors:** M. Aaboud, G. Aad, B. Abbott, J. Abdallah, O. Abdinov, B. Abeloos, S. H. Abidi, O. S. AbouZeid, N. L. Abraham, H. Abramowicz, H. Abreu, R. Abreu, Y. Abulaiti, B. S. Acharya, S. Adachi, L. Adamczyk, J. Adelman, M. Adersberger, T. Adye, A. A. Affolder, T. Agatonovic-Jovin, C. Agheorghiesei, J. A. Aguilar-Saavedra, S. P. Ahlen, F. Ahmadov, G. Aielli, S. Akatsuka, H. Akerstedt, T. P. A. Åkesson, A. V. Akimov, G. L. Alberghi, J. Albert, P. Albicocco, M. J. Alconada Verzini, M. Aleksa, I. N. Aleksandrov, C. Alexa, G. Alexander, T. Alexopoulos, M. Alhroob, B. Ali, M. Aliev, G. Alimonti, J. Alison, S. P. Alkire, B. M. M. Allbrooke, B. W. Allen, P. P. Allport, A. Aloisio, A. Alonso, F. Alonso, C. Alpigiani, A. A. Alshehri, M. Alstaty, B. Alvarez Gonzalez, D. Álvarez Piqueras, M. G. Alviggi, B. T. Amadio, Y. Amaral Coutinho, C. Amelung, D. Amidei, S. P. Amor Dos Santos, A. Amorim, S. Amoroso, G. Amundsen, C. Anastopoulos, L. S. Ancu, N. Andari, T. Andeen, C. F. Anders, J. K. Anders, K. J. Anderson, A. Andreazza, V. Andrei, S. Angelidakis, I. Angelozzi, A. Angerami, A. V. Anisenkov, N. Anjos, A. Annovi, C. Antel, M. Antonelli, A. Antonov, D. J. Antrim, F. Anulli, M. Aoki, L. Aperio Bella, G. Arabidze, Y. Arai, J. P. Araque, V. Araujo Ferraz, A. T. H. Arce, R. E. Ardell, F. A. Arduh, J.-F. Arguin, S. Argyropoulos, M. Arik, A. J. Armbruster, L. J. Armitage, O. Arnaez, H. Arnold, M. Arratia, O. Arslan, A. Artamonov, G. Artoni, S. Artz, S. Asai, N. Asbah, A. Ashkenazi, L. Asquith, K. Assamagan, R. Astalos, M. Atkinson, N. B. Atlay, L. Aubry, K. Augsten, G. Avolio, B. Axen, M. K. Ayoub, G. Azuelos, A. E. Baas, M. J. Baca, H. Bachacou, K. Bachas, M. Backes, M. Backhaus, P. Bagnaia, H. Bahrasemani, J. T. Baines, M. Bajic, O. K. Baker, E. M. Baldin, P. Balek, F. Balli, W. K. Balunas, E. Banas, Sw. Banerjee, A. A. E. Bannoura, L. Barak, E. L. Barberio, D. Barberis, M. Barbero, T. Barillari, M.-S. Barisits, T. Barklow, N. Barlow, S. L. Barnes, B. M. Barnett, R. M. Barnett, Z. Barnovska-Blenessy, A. Baroncelli, G. Barone, A. J. Barr, L. Barranco Navarro, F. Barreiro, J. Barreiro Guimarães da Costa, R. Bartoldus, A. E. Barton, P. Bartos, A. Basalaev, A. Bassalat, R. L. Bates, S. J. Batista, J. R. Batley, M. Battaglia, M. Bauce, F. Bauer, H. S. Bawa, J. B. Beacham, M. D. Beattie, T. Beau, P. H. Beauchemin, P. Bechtle, H. P. Beck, K. Becker, M. Becker, M. Beckingham, C. Becot, A. J. Beddall, A. Beddall, V. A. Bednyakov, M. Bedognetti, C. P. Bee, T. A. Beermann, M. Begalli, M. Begel, J. K. Behr, A. S. Bell, G. Bella, L. Bellagamba, A. Bellerive, M. Bellomo, K. Belotskiy, O. Beltramello, N. L. Belyaev, O. Benary, D. Benchekroun, M. Bender, K. Bendtz, N. Benekos, Y. Benhammou, E. Benhar Noccioli, J. Benitez, D. P. Benjamin, M. Benoit, J. R. Bensinger, S. Bentvelsen, L. Beresford, M. Beretta, D. Berge, E. Bergeaas Kuutmann, N. Berger, J. Beringer, S. Berlendis, N. R. Bernard, G. Bernardi, C. Bernius, F. U. Bernlochner, T. Berry, P. Berta, C. Bertella, G. Bertoli, F. Bertolucci, I. A. Bertram, C. Bertsche, D. Bertsche, G. J. Besjes, O. Bessidskaia Bylund, M. Bessner, N. Besson, C. Betancourt, A. Bethani, S. Bethke, A. J. Bevan, J. Beyer, R. M. Bianchi, O. Biebel, D. Biedermann, R. Bielski, N. V. Biesuz, M. Biglietti, J. Bilbao De Mendizabal, T. R. V. Billoud, H. Bilokon, M. Bindi, A. Bingul, C. Bini, S. Biondi, T. Bisanz, C. Bittrich, D. M. Bjergaard, C. W. Black, J. E. Black, K. M. Black, R. E. Blair, T. Blazek, I. Bloch, C. Blocker, A. Blue, W. Blum, U. Blumenschein, S. Blunier, G. J. Bobbink, V. S. Bobrovnikov, S. S. Bocchetta, A. Bocci, C. Bock, M. Boehler, D. Boerner, D. Bogavac, A. G. Bogdanchikov, C. Bohm, V. Boisvert, P. Bokan, T. Bold, A. S. Boldyrev, A. E. Bolz, M. Bomben, M. Bona, M. Boonekamp, A. Borisov, G. Borissov, J. Bortfeldt, D. Bortoletto, V. Bortolotto, D. Boscherini, M. Bosman, J. D. Bossio Sola, J. Boudreau, J. Bouffard, E. V. Bouhova-Thacker, D. Boumediene, C. Bourdarios, S. K. Boutle, A. Boveia, J. Boyd, I. R. Boyko, J. Bracinik, A. Brandt, G. Brandt, O. Brandt, U. Bratzler, B. Brau, J. E. Brau, W. D. Breaden Madden, K. Brendlinger, A. J. Brennan, L. Brenner, R. Brenner, S. Bressler, D. L. Briglin, T. M. Bristow, D. Britton, D. Britzger, F. M. Brochu, I. Brock, R. Brock, G. Brooijmans, T. Brooks, W. K. Brooks, J. Brosamer, E. Brost, J. H Broughton, P. A. Bruckman de Renstrom, D. Bruncko, A. Bruni, G. Bruni, L. S. Bruni, BH Brunt, M. Bruschi, N. Bruscino, P. Bryant, L. Bryngemark, T. Buanes, Q. Buat, P. Buchholz, A. G. Buckley, I. A. Budagov, F. Buehrer, M. K. Bugge, O. Bulekov, D. Bullock, T. J. Burch, H. Burckhart, S. Burdin, C. D. Burgard, A. M. Burger, B. Burghgrave, K. Burka, S. Burke, I. Burmeister, J. T. P. Burr, E. Busato, D. Büscher, V. Büscher, P. Bussey, J. M. Butler, C. M. Buttar, J. M. Butterworth, P. Butti, W. Buttinger, A. Buzatu, A. R. Buzykaev, S. Cabrera Urbán, D. Caforio, V. M. Cairo, O. Cakir, N. Calace, P. Calafiura, A. Calandri, G. Calderini, P. Calfayan, G. Callea, L. P. Caloba, S. Calvente Lopez, D. Calvet, S. Calvet, T. P. Calvet, R. Camacho Toro, S. Camarda, P. Camarri, D. Cameron, R. Caminal Armadans, C. Camincher, S. Campana, M. Campanelli, A. Camplani, A. Campoverde, V. Canale, M. Cano Bret, J. Cantero, T. Cao, M. D. M. Capeans Garrido, I. Caprini, M. Caprini, M. Capua, R. M. Carbone, R. Cardarelli, F. Cardillo, I. Carli, T. Carli, G. Carlino, B. T. Carlson, L. Carminati, R. M. D. Carney, S. Caron, E. Carquin, S. Carrá, G. D. Carrillo-Montoya, J. Carvalho, D. Casadei, M. P. Casado, M. Casolino, D. W. Casper, R. Castelijn, V. Castillo Gimenez, N. F. Castro, A. Catinaccio, J. R. Catmore, A. Cattai, J. Caudron, V. Cavaliere, E. Cavallaro, D. Cavalli, M. Cavalli-Sforza, V. Cavasinni, E. Celebi, F. Ceradini, L. Cerda Alberich, A. S. Cerqueira, A. Cerri, L. Cerrito, F. Cerutti, A. Cervelli, S. A. Cetin, A. Chafaq, D. Chakraborty, S. K. Chan, W. S. Chan, Y. L. Chan, P. Chang, J. D. Chapman, D. G. Charlton, C. C. Chau, C. A. Chavez Barajas, S. Che, S. Cheatham, A. Chegwidden, S. Chekanov, S. V. Chekulaev, G. A. Chelkov, M. A. Chelstowska, C. Chen, H. Chen, S. Chen, S. Chen, X. Chen, Y. Chen, H. C. Cheng, H. J. Cheng, A. Cheplakov, E. Cheremushkina, R. Cherkaoui El Moursli, V. Chernyatin, E. Cheu, L. Chevalier, V. Chiarella, G. Chiarelli, G. Chiodini, A. S. Chisholm, A. Chitan, Y. H. Chiu, M. V. Chizhov, K. Choi, A. R. Chomont, S. Chouridou, V. Christodoulou, D. Chromek-Burckhart, M. C. Chu, J. Chudoba, A. J. Chuinard, J. J. Chwastowski, L. Chytka, A. K. Ciftci, D. Cinca, V. Cindro, I. A. Cioara, C. Ciocca, A. Ciocio, F. Cirotto, Z. H. Citron, M. Citterio, M. Ciubancan, A. Clark, B. L. Clark, M. R. Clark, P. J. Clark, R. N. Clarke, C. Clement, Y. Coadou, M. Cobal, A. Coccaro, J. Cochran, L. Colasurdo, B. Cole, A. P. Colijn, J. Collot, T. Colombo, P. Conde Muiño, E. Coniavitis, S. H. Connell, I. A. Connelly, S. Constantinescu, G. Conti, F. Conventi, M. Cooke, A. M. Cooper-Sarkar, F. Cormier, K. J. R. Cormier, M. Corradi, F. Corriveau, A. Cortes-Gonzalez, G. Cortiana, G. Costa, M. J. Costa, D. Costanzo, G. Cottin, G. Cowan, B. E. Cox, K. Cranmer, S. J. Crawley, R. A. Creager, G. Cree, S. Crépé-Renaudin, F. Crescioli, W. A. Cribbs, M. Cristinziani, V. Croft, G. Crosetti, A. Cueto, T. Cuhadar Donszelmann, A. R. Cukierman, J. Cummings, M. Curatolo, J. Cúth, H. Czirr, P. Czodrowski, G. D’amen, S. D’Auria, L. D’eramo, M. D’Onofrio, M. J. Da Cunha Sargedas De Sousa, C. Da Via, W. Dabrowski, T. Dado, T. Dai, O. Dale, F. Dallaire, C. Dallapiccola, M. Dam, J. R. Dandoy, M. F. Daneri, N. P. Dang, A. C. Daniells, N. S. Dann, M. Danninger, M. Dano Hoffmann, V. Dao, G. Darbo, S. Darmora, J. Dassoulas, A. Dattagupta, T. Daubney, W. Davey, C. David, T. Davidek, M. Davies, D. R. Davis, P. Davison, E. Dawe, I. Dawson, K. De, R. de Asmundis, A. De Benedetti, S. De Castro, S. De Cecco, N. De Groot, P. de Jong, H. De la Torre, F. De Lorenzi, A. De Maria, D. De Pedis, A. De Salvo, U. De Sanctis, A. De Santo, K. De Vasconcelos Corga, J. B. De Vivie De Regie, W. J. Dearnaley, R. Debbe, C. Debenedetti, D. V. Dedovich, N. Dehghanian, I. Deigaard, M. Del Gaudio, J. Del Peso, T. Del Prete, D. Delgove, F. Deliot, C. M. Delitzsch, A. Dell’Acqua, L. Dell’Asta, M. Dell’Orso, M. Della Pietra, D. della Volpe, M. Delmastro, C. Delporte, P. A. Delsart, D. A. DeMarco, S. Demers, M. Demichev, A. Demilly, S. P. Denisov, D. Denysiuk, D. Derendarz, J. E. Derkaoui, F. Derue, P. Dervan, K. Desch, C. Deterre, K. Dette, M. R. Devesa, P. O. Deviveiros, A. Dewhurst, S. Dhaliwal, F. A. Di Bello, A. Di Ciaccio, L. Di Ciaccio, W. K. Di Clemente, C. Di Donato, A. Di Girolamo, B. Di Girolamo, B. Di Micco, R. Di Nardo, K. F. Di Petrillo, A. Di Simone, R. Di Sipio, D. Di Valentino, C. Diaconu, M. Diamond, F. A. Dias, M. A. Diaz, E. B. Diehl, J. Dietrich, S. Díez Cornell, A. Dimitrievska, J. Dingfelder, P. Dita, S. Dita, F. Dittus, F. Djama, T. Djobava, J. I. Djuvsland, M. A. B. do Vale, D. Dobos, M. Dobre, C. Doglioni, J. Dolejsi, Z. Dolezal, M. Donadelli, S. Donati, P. Dondero, J. Donini, J. Dopke, A. Doria, M. T. Dova, A. T. Doyle, E. Drechsler, M. Dris, Y. Du, J. Duarte-Campderros, A. Dubreuil, E. Duchovni, G. Duckeck, A. Ducourthial, O. A. Ducu, D. Duda, A. Dudarev, A. Chr. Dudder, E. M. Duffield, L. Duflot, M. Dührssen, M. Dumancic, A. E. Dumitriu, A. K. Duncan, M. Dunford, H. Duran Yildiz, M. Düren, A. Durglishvili, D. Duschinger, B. Dutta, M. Dyndal, C. Eckardt, K. M. Ecker, R. C. Edgar, T. Eifert, G. Eigen, K. Einsweiler, T. Ekelof, M. El Kacimi, R. El Kosseifi, V. Ellajosyula, M. Ellert, S. Elles, F. Ellinghaus, A. A. Elliot, N. Ellis, J. Elmsheuser, M. Elsing, D. Emeliyanov, Y. Enari, O. C. Endner, J. S. Ennis, J. Erdmann, A. Ereditato, G. Ernis, M. Ernst, S. Errede, M. Escalier, C. Escobar, B. Esposito, O. Estrada Pastor, A. I. Etienvre, E. Etzion, H. Evans, A. Ezhilov, M. Ezzi, F. Fabbri, L. Fabbri, G. Facini, R. M. Fakhrutdinov, S. Falciano, R. J. Falla, J. Faltova, Y. Fang, M. Fanti, A. Farbin, A. Farilla, C. Farina, E. M. Farina, T. Farooque, S. Farrell, S. M. Farrington, P. Farthouat, F. Fassi, P. Fassnacht, D. Fassouliotis, M. Faucci Giannelli, A. Favareto, W. J. Fawcett, L. Fayard, O. L. Fedin, W. Fedorko, S. Feigl, L. Feligioni, C. Feng, E. J. Feng, H. Feng, M. J. Fenton, A. B. Fenyuk, L. Feremenga, P. Fernandez Martinez, S. Fernandez Perez, J. Ferrando, A. Ferrari, P. Ferrari, R. Ferrari, D. E. Ferreira de Lima, A. Ferrer, D. Ferrere, C. Ferretti, F. Fiedler, A. Filipčič, M. Filipuzzi, F. Filthaut, M. Fincke-Keeler, K. D. Finelli, M. C. N. Fiolhais, L. Fiorini, A. Fischer, C. Fischer, J. Fischer, W. C. Fisher, N. Flaschel, I. Fleck, P. Fleischmann, R. R. M. Fletcher, T. Flick, B. M. Flierl, L. R. Flores Castillo, M. J. Flowerdew, G. T. Forcolin, A. Formica, F. A. Förster, A. Forti, A. G. Foster, D. Fournier, H. Fox, S. Fracchia, P. Francavilla, M. Franchini, S. Franchino, D. Francis, L. Franconi, M. Franklin, M. Frate, M. Fraternali, D. Freeborn, S. M. Fressard-Batraneanu, B. Freund, D. Froidevaux, J. A. Frost, C. Fukunaga, T. Fusayasu, J. Fuster, C. Gabaldon, O. Gabizon, A. Gabrielli, A. Gabrielli, G. P. Gach, S. Gadatsch, S. Gadomski, G. Gagliardi, L. G. Gagnon, C. Galea, B. Galhardo, E. J. Gallas, B. J. Gallop, P. Gallus, G. Galster, K. K. Gan, S. Ganguly, Y. Gao, Y. S. Gao, F. M. Garay Walls, C. García, J. E. García Navarro, M. Garcia-Sciveres, R. W. Gardner, N. Garelli, V. Garonne, A. Gascon Bravo, K. Gasnikova, C. Gatti, A. Gaudiello, G. Gaudio, I. L. Gavrilenko, C. Gay, G. Gaycken, E. N. Gazis, C. N. P. Gee, J. Geisen, M. Geisen, M. P. Geisler, K. Gellerstedt, C. Gemme, M. H. Genest, C. Geng, S. Gentile, C. Gentsos, S. George, D. Gerbaudo, A. Gershon, G. Geßner, S. Ghasemi, M. Ghneimat, B. Giacobbe, S. Giagu, P. Giannetti, S. M. Gibson, M. Gignac, M. Gilchriese, D. Gillberg, G. Gilles, D. M. Gingrich, N. Giokaris, M. P. Giordani, F. M. Giorgi, P. F. Giraud, P. Giromini, D. Giugni, F. Giuli, C. Giuliani, M. Giulini, B. K. Gjelsten, S. Gkaitatzis, I. Gkialas, E. L. Gkougkousis, P. Gkountoumis, L. K. Gladilin, C. Glasman, J. Glatzer, P. C. F. Glaysher, A. Glazov, M. Goblirsch-Kolb, J. Godlewski, S. Goldfarb, T. Golling, D. Golubkov, A. Gomes, R. Gonçalo, R. Goncalves Gama, J. Goncalves Pinto Firmino Da Costa, G. Gonella, L. Gonella, A. Gongadze, S. González de la Hoz, S. Gonzalez-Sevilla, L. Goossens, P. A. Gorbounov, H. A. Gordon, I. Gorelov, B. Gorini, E. Gorini, A. Gorišek, A. T. Goshaw, C. Gössling, M. I. Gostkin, C. A. Gottardo, C. R. Goudet, D. Goujdami, A. G. Goussiou, N. Govender, E. Gozani, L. Graber, I. Grabowska-Bold, P. O. J. Gradin, J. Gramling, E. Gramstad, S. Grancagnolo, V. Gratchev, P. M. Gravila, C. Gray, H. M. Gray, Z. D. Greenwood, C. Grefe, K. Gregersen, I. M. Gregor, P. Grenier, K. Grevtsov, J. Griffiths, A. A. Grillo, K. Grimm, S. Grinstein, Ph. Gris, J.-F. Grivaz, S. Groh, E. Gross, J. Grosse-Knetter, G. C. Grossi, Z. J. Grout, A. Grummer, L. Guan, W. Guan, J. Guenther, F. Guescini, D. Guest, O. Gueta, B. Gui, E. Guido, T. Guillemin, S. Guindon, U. Gul, C. Gumpert, J. Guo, W. Guo, Y. Guo, R. Gupta, S. Gupta, G. Gustavino, P. Gutierrez, N. G. Gutierrez Ortiz, C. Gutschow, C. Guyot, M. P. Guzik, C. Gwenlan, C. B. Gwilliam, A. Haas, C. Haber, H. K. Hadavand, N. Haddad, A. Hadef, S. Hageböck, M. Hagihara, H. Hakobyan, M. Haleem, J. Haley, G. Halladjian, G. D. Hallewell, K. Hamacher, P. Hamal, K. Hamano, A. Hamilton, G. N. Hamity, P. G. Hamnett, L. Han, S. Han, K. Hanagaki, K. Hanawa, M. Hance, B. Haney, P. Hanke, J. B. Hansen, J. D. Hansen, M. C. Hansen, P. H. Hansen, K. Hara, A. S. Hard, T. Harenberg, F. Hariri, S. Harkusha, R. D. Harrington, P. F. Harrison, N. M. Hartmann, M. Hasegawa, Y. Hasegawa, A. Hasib, S. Hassani, S. Haug, R. Hauser, L. Hauswald, L. B. Havener, M. Havranek, C. M. Hawkes, R. J. Hawkings, D. Hayakawa, D. Hayden, C. P. Hays, J. M. Hays, H. S. Hayward, S. J. Haywood, S. J. Head, T. Heck, V. Hedberg, L. Heelan, K. K. Heidegger, S. Heim, T. Heim, B. Heinemann, J. J. Heinrich, L. Heinrich, C. Heinz, J. Hejbal, L. Helary, A. Held, S. Hellman, C. Helsens, R. C. W. Henderson, Y. Heng, S. Henkelmann, A. M. Henriques Correia, S. Henrot-Versille, G. H. Herbert, H. Herde, V. Herget, Y. Hernández Jiménez, G. Herten, R. Hertenberger, L. Hervas, T. C. Herwig, G. G. Hesketh, N. P. Hessey, J. W. Hetherly, S. Higashino, E. Higón-Rodriguez, E. Hill, J. C. Hill, K. H. Hiller, S. J. Hillier, M. Hils, I. Hinchliffe, M. Hirose, D. Hirschbuehl, B. Hiti, O. Hladik, X. Hoad, J. Hobbs, N. Hod, M. C. Hodgkinson, P. Hodgson, A. Hoecker, M. R. Hoeferkamp, F. Hoenig, D. Hohn, T. R. Holmes, M. Homann, S. Honda, T. Honda, T. M. Hong, B. H. Hooberman, W. H. Hopkins, Y. Horii, A. J. Horton, J.-Y. Hostachy, S. Hou, A. Hoummada, J. Howarth, J. Hoya, M. Hrabovsky, J. Hrdinka, I. Hristova, J. Hrivnac, T. Hryn’ova, A. Hrynevich, P. J. Hsu, S.-C. Hsu, Q. Hu, S. Hu, Y. Huang, Z. Hubacek, F. Hubaut, F. Huegging, T. B. Huffman, E. W. Hughes, G. Hughes, M. Huhtinen, P. Huo, N. Huseynov, J. Huston, J. Huth, G. Iacobucci, G. Iakovidis, I. Ibragimov, L. Iconomidou-Fayard, Z. Idrissi, P. Iengo, O. Igonkina, T. Iizawa, Y. Ikegami, M. Ikeno, Y. Ilchenko, D. Iliadis, N. Ilic, G. Introzzi, P. Ioannou, M. Iodice, K. Iordanidou, V. Ippolito, M. F. Isacson, N. Ishijima, M. Ishino, M. Ishitsuka, C. Issever, S. Istin, F. Ito, J. M. Iturbe Ponce, R. Iuppa, H. Iwasaki, J. M. Izen, V. Izzo, S. Jabbar, P. Jackson, R. M. Jacobs, V. Jain, K. B. Jakobi, K. Jakobs, S. Jakobsen, T. Jakoubek, D. O. Jamin, D. K. Jana, R. Jansky, J. Janssen, M. Janus, P. A. Janus, G. Jarlskog, N. Javadov, T. Javůrek, M. Javurkova, F. Jeanneau, L. Jeanty, J. Jejelava, A. Jelinskas, P. Jenni, C. Jeske, S. Jézéquel, H. Ji, J. Jia, H. Jiang, Y. Jiang, Z. Jiang, S. Jiggins, J. Jimenez Pena, S. Jin, A. Jinaru, O. Jinnouchi, H. Jivan, P. Johansson, K. A. Johns, C. A. Johnson, W. J. Johnson, K. Jon-And, R. W. L. Jones, S. D. Jones, S. Jones, T. J. Jones, J. Jongmanns, P. M. Jorge, J. Jovicevic, X. Ju, A. Juste Rozas, M. K. Köhler, A. Kaczmarska, M. Kado, H. Kagan, M. Kagan, S. J. Kahn, T. Kaji, E. Kajomovitz, C. W. Kalderon, A. Kaluza, S. Kama, A. Kamenshchikov, N. Kanaya, L. Kanjir, V. A. Kantserov, J. Kanzaki, B. Kaplan, L. S. Kaplan, D. Kar, K. Karakostas, N. Karastathis, M. J. Kareem, E. Karentzos, S. N. Karpov, Z. M. Karpova, K. Karthik, V. Kartvelishvili, A. N. Karyukhin, K. Kasahara, L. Kashif, R. D. Kass, A. Kastanas, Y. Kataoka, C. Kato, A. Katre, J. Katzy, K. Kawade, K. Kawagoe, T. Kawamoto, G. Kawamura, E. F. Kay, V. F. Kazanin, R. Keeler, R. Kehoe, J. S. Keller, J. J. Kempster, J Kendrick, H. Keoshkerian, O. Kepka, B. P. Kerševan, S. Kersten, R. A. Keyes, M. Khader, F. Khalil-zada, A. Khanov, A. G. Kharlamov, T. Kharlamova, A. Khodinov, T. J. Khoo, V. Khovanskiy, E. Khramov, J. Khubua, S. Kido, C. R. Kilby, H. Y. Kim, S. H. Kim, Y. K. Kim, N. Kimura, O. M. Kind, B. T. King, D. Kirchmeier, J. Kirk, A. E. Kiryunin, T. Kishimoto, D. Kisielewska, K. Kiuchi, O. Kivernyk, E. Kladiva, T. Klapdor-Kleingrothaus, M. H. Klein, M. Klein, U. Klein, K. Kleinknecht, P. Klimek, A. Klimentov, R. Klingenberg, T. Klingl, T. Klioutchnikova, E.-E. Kluge, P. Kluit, S. Kluth, E. Kneringer, E. B. F. G. Knoops, A. Knue, A. Kobayashi, D. Kobayashi, T. Kobayashi, M. Kobel, M. Kocian, P. Kodys, T. Koffas, E. Koffeman, N. M. Köhler, T. Koi, M. Kolb, I. Koletsou, A. A. Komar, Y. Komori, T. Kondo, N. Kondrashova, K. Köneke, A. C. König, T. Kono, R. Konoplich, N. Konstantinidis, R. Kopeliansky, S. Koperny, A. K. Kopp, K. Korcyl, K. Kordas, A. Korn, A. A. Korol, I. Korolkov, E. V. Korolkova, O. Kortner, S. Kortner, T. Kosek, V. V. Kostyukhin, A. Kotwal, A. Koulouris, A. Kourkoumeli-Charalampidi, C. Kourkoumelis, E. Kourlitis, V. Kouskoura, A. B. Kowalewska, R. Kowalewski, T. Z. Kowalski, C. Kozakai, W. Kozanecki, A. S. Kozhin, V. A. Kramarenko, G. Kramberger, D. Krasnopevtsev, M. W. Krasny, A. Krasznahorkay, D. Krauss, J. A. Kremer, J. Kretzschmar, K. Kreutzfeldt, P. Krieger, K. Krizka, K. Kroeninger, H. Kroha, J. Kroll, J. Kroll, J. Kroseberg, J. Krstic, U. Kruchonak, H. Krüger, N. Krumnack, M. C. Kruse, T. Kubota, H. Kucuk, S. Kuday, J. T. Kuechler, S. Kuehn, A. Kugel, F. Kuger, T. Kuhl, V. Kukhtin, R. Kukla, Y. Kulchitsky, S. Kuleshov, Y. P. Kulinich, M. Kuna, T. Kunigo, A. Kupco, T. Kupfer, O. Kuprash, H. Kurashige, L. L. Kurchaninov, Y. A. Kurochkin, M. G. Kurth, V. Kus, E. S. Kuwertz, M. Kuze, J. Kvita, T. Kwan, D. Kyriazopoulos, A. La Rosa, J. L. La Rosa Navarro, L. La Rotonda, C. Lacasta, F. Lacava, J. Lacey, H. Lacker, D. Lacour, E. Ladygin, R. Lafaye, B. Laforge, T. Lagouri, S. Lai, S. Lammers, W. Lampl, E. Lançon, U. Landgraf, M. P. J. Landon, M. C. Lanfermann, V. S. Lang, J. C. Lange, R. J. Langenberg, A. J. Lankford, F. Lanni, K. Lantzsch, A. Lanza, A. Lapertosa, S. Laplace, J. F. Laporte, T. Lari, F. Lasagni Manghi, M. Lassnig, P. Laurelli, W. Lavrijsen, A. T. Law, P. Laycock, T. Lazovich, M. Lazzaroni, B. Le, O. Le Dortz, E. Le Guirriec, E. P. Le Quilleuc, M. LeBlanc, T. LeCompte, F. Ledroit-Guillon, C. A. Lee, G. R. Lee, S. C. Lee, L. Lee, B. Lefebvre, G. Lefebvre, M. Lefebvre, F. Legger, C. Leggett, A. Lehan, G. Lehmann Miotto, X. Lei, W. A. Leight, M. A. L. Leite, R. Leitner, D. Lellouch, B. Lemmer, K. J. C. Leney, T. Lenz, B. Lenzi, R. Leone, S. Leone, C. Leonidopoulos, G. Lerner, C. Leroy, A. A. J. Lesage, C. G. Lester, M. Levchenko, J. Levêque, D. Levin, L. J. Levinson, M. Levy, D. Lewis, B. Li, C. Li, H. Li, L. Li, Q. Li, S. Li, X. Li, Y. Li, Z. Liang, B. Liberti, A. Liblong, K. Lie, J. Liebal, W. Liebig, A. Limosani, S. C. Lin, T. H. Lin, B. E. Lindquist, A. E. Lionti, E. Lipeles, A. Lipniacka, M. Lisovyi, T. M. Liss, A. Lister, A. M. Litke, B. Liu, H. Liu, H. Liu, J. K. K. Liu, J. Liu, J. B. Liu, K. Liu, L. Liu, M. Liu, Y. L. Liu, Y. Liu, M. Livan, A. Lleres, J. Llorente Merino, S. L. Lloyd, C. Y. Lo, F. Lo Sterzo, E. M. Lobodzinska, P. Loch, F. K. Loebinger, A. Loesle, K. M. Loew, A. Loginov, T. Lohse, K. Lohwasser, M. Lokajicek, B. A. Long, J. D. Long, R. E. Long, L. Longo, K. A. Looper, J. A. Lopez, D. Lopez Mateos, I. Lopez Paz, A. Lopez Solis, J. Lorenz, N. Lorenzo Martinez, M. Losada, P. J. Lösel, X. Lou, A. Lounis, J. Love, P. A. Love, H. Lu, N. Lu, Y. J. Lu, H. J. Lubatti, C. Luci, A. Lucotte, C. Luedtke, F. Luehring, W. Lukas, L. Luminari, O. Lundberg, B. Lund-Jensen, P. M. Luzi, D. Lynn, R. Lysak, E. Lytken, V. Lyubushkin, H. Ma, L. L. Ma, Y. Ma, G. Maccarrone, A. Macchiolo, C. M. Macdonald, B. Maček, J. Machado Miguens, D. Madaffari, R. Madar, W. F. Mader, A. Madsen, J. Maeda, S. Maeland, T. Maeno, A. S. Maevskiy, E. Magradze, J. Mahlstedt, C. Maiani, C. Maidantchik, A. A. Maier, T. Maier, A. Maio, O. Majersky, S. Majewski, Y. Makida, N. Makovec, B. Malaescu, Pa. Malecki, V. P. Maleev, F. Malek, U. Mallik, D. Malon, C. Malone, S. Maltezos, S. Malyukov, J. Mamuzic, G. Mancini, L. Mandelli, I. Mandić, J. Maneira, L. Manhaes de Andrade Filho, J. Manjarres Ramos, A. Mann, A. Manousos, B. Mansoulie, J. D. Mansour, R. Mantifel, M. Mantoani, S. Manzoni, L. Mapelli, G. Marceca, L. March, L. Marchese, G. Marchiori, M. Marcisovsky, M. Marjanovic, D. E. Marley, F. Marroquim, S. P. Marsden, Z. Marshall, M. U. F Martensson, S. Marti-Garcia, C. B. Martin, T. A. Martin, V. J. Martin, B. Martin dit Latour, M. Martinez, V. I. Martinez Outschoorn, S. Martin-Haugh, V. S. Martoiu, A. C. Martyniuk, A. Marzin, L. Masetti, T. Mashimo, R. Mashinistov, J. Masik, A. L. Maslennikov, L. Massa, P. Mastrandrea, A. Mastroberardino, T. Masubuchi, P. Mättig, J. Maurer, S. J. Maxfield, D. A. Maximov, R. Mazini, I. Maznas, S. M. Mazza, N. C. Mc Fadden, G. Mc Goldrick, S. P. Mc Kee, A. McCarn, R. L. McCarthy, T. G. McCarthy, L. I. McClymont, E. F. McDonald, J. A. Mcfayden, G. Mchedlidze, S. J. McMahon, P. C. McNamara, R. A. McPherson, S. Meehan, T. J. Megy, S. Mehlhase, A. Mehta, T. Meideck, K. Meier, B. Meirose, D. Melini, B. R. Mellado Garcia, J. D. Mellenthin, M. Melo, F. Meloni, S. B. Menary, L. Meng, X. T. Meng, A. Mengarelli, S. Menke, E. Meoni, S. Mergelmeyer, P. Mermod, L. Merola, C. Meroni, F. S. Merritt, A. Messina, J. Metcalfe, A. S. Mete, C. Meyer, J.-P. Meyer, J. Meyer, H. Meyer Zu Theenhausen, F. Miano, R. P. Middleton, S. Miglioranzi, L. Mijović, G. Mikenberg, M. Mikestikova, M. Mikuž, M. Milesi, A. Milic, D. W. Miller, C. Mills, A. Milov, D. A. Milstead, A. A. Minaenko, Y. Minami, I. A. Minashvili, A. I. Mincer, B. Mindur, M. Mineev, Y. Minegishi, Y. Ming, L. M. Mir, K. P. Mistry, T. Mitani, J. Mitrevski, V. A. Mitsou, A. Miucci, P. S. Miyagawa, A. Mizukami, J. U. Mjörnmark, T. Mkrtchyan, M. Mlynarikova, T. Moa, K. Mochizuki, P. Mogg, S. Mohapatra, S. Molander, R. Moles-Valls, R. Monden, M. C. Mondragon, K. Mönig, J. Monk, E. Monnier, A. Montalbano, J. Montejo Berlingen, F. Monticelli, S. Monzani, R. W. Moore, N. Morange, D. Moreno, M. Moreno Llácer, P. Morettini, S. Morgenstern, D. Mori, T. Mori, M. Morii, M. Morinaga, V. Morisbak, A. K. Morley, G. Mornacchi, J. D. Morris, L. Morvaj, P. Moschovakos, M. Mosidze, H. J. Moss, J. Moss, K. Motohashi, R. Mount, E. Mountricha, E. J. W. Moyse, S. Muanza, R. D. Mudd, F. Mueller, J. Mueller, R. S. P. Mueller, D. Muenstermann, P. Mullen, G. A. Mullier, F. J. Munoz Sanchez, W. J. Murray, H. Musheghyan, M. Muškinja, A. G. Myagkov, M. Myska, B. P. Nachman, O. Nackenhorst, K. Nagai, R. Nagai, K. Nagano, Y. Nagasaka, K. Nagata, M. Nagel, E. Nagy, A. M. Nairz, Y. Nakahama, K. Nakamura, T. Nakamura, I. Nakano, R. F. Naranjo Garcia, R. Narayan, D. I. Narrias Villar, I. Naryshkin, T. Naumann, G. Navarro, R. Nayyar, H. A. Neal, P. Yu. Nechaeva, T. J. Neep, A. Negri, M. Negrini, S. Nektarijevic, C. Nellist, A. Nelson, M. E. Nelson, S. Nemecek, P. Nemethy, M. Nessi, M. S. Neubauer, M. Neumann, P. R. Newman, T. Y. Ng, T. Nguyen Manh, R. B. Nickerson, R. Nicolaidou, J. Nielsen, V. Nikolaenko, I. Nikolic-Audit, K. Nikolopoulos, J. K. Nilsen, P. Nilsson, Y. Ninomiya, A. Nisati, N. Nishu, R. Nisius, I. Nitsche, T. Nobe, Y. Noguchi, M. Nomachi, I. Nomidis, M. A. Nomura, T. Nooney, M. Nordberg, N. Norjoharuddeen, O. Novgorodova, S. Nowak, M. Nozaki, L. Nozka, K. Ntekas, E. Nurse, F. Nuti, K. O’connor, D. C. O’Neil, A. A. O’Rourke, V. O’Shea, F. G. Oakham, H. Oberlack, T. Obermann, J. Ocariz, A. Ochi, I. Ochoa, J. P. Ochoa-Ricoux, S. Oda, S. Odaka, H. Ogren, A. Oh, S. H. Oh, C. C. Ohm, H. Ohman, H. Oide, H. Okawa, Y. Okumura, T. Okuyama, A. Olariu, L. F. Oleiro Seabra, S. A. Olivares Pino, D. Oliveira Damazio, A. Olszewski, J. Olszowska, A. Onofre, K. Onogi, P. U. E. Onyisi, M. J. Oreglia, Y. Oren, D. Orestano, N. Orlando, R. S. Orr, B. Osculati, R. Ospanov, G. Otero y Garzon, H. Otono, M. Ouchrif, F. Ould-Saada, A. Ouraou, K. P. Oussoren, Q. Ouyang, M. Owen, R. E. Owen, V. E. Ozcan, N. Ozturk, K. Pachal, A. Pacheco Pages, L. Pacheco Rodriguez, C. Padilla Aranda, S. Pagan Griso, M. Paganini, F. Paige, G. Palacino, S. Palazzo, S. Palestini, M. Palka, D. Pallin, E. St. Panagiotopoulou, I. Panagoulias, C. E. Pandini, J. G. Panduro Vazquez, P. Pani, S. Panitkin, D. Pantea, L. Paolozzi, Th. D. Papadopoulou, K. Papageorgiou, A. Paramonov, D. Paredes Hernandez, A. J. Parker, M. A. Parker, K. A. Parker, F. Parodi, J. A. Parsons, U. Parzefall, V. R. Pascuzzi, J. M. Pasner, E. Pasqualucci, S. Passaggio, Fr. Pastore, S. Pataraia, J. R. Pater, T. Pauly, B. Pearson, S. Pedraza Lopez, R. Pedro, S. V. Peleganchuk, O. Penc, C. Peng, H. Peng, J. Penwell, B. S. Peralva, M. M. Perego, D. V. Perepelitsa, L. Perini, H. Pernegger, S. Perrella, R. Peschke, V. D. Peshekhonov, K. Peters, R. F. Y. Peters, B. A. Petersen, T. C. Petersen, E. Petit, A. Petridis, C. Petridou, P. Petroff, E. Petrolo, M. Petrov, F. Petrucci, N. E. Pettersson, A. Peyaud, R. Pezoa, F. H. Phillips, P. W. Phillips, G. Piacquadio, E. Pianori, A. Picazio, E. Piccaro, M. A. Pickering, R. Piegaia, J. E. Pilcher, A. D. Pilkington, A. W. J. Pin, M. Pinamonti, J. L. Pinfold, H. Pirumov, M. Pitt, L. Plazak, M.-A. Pleier, V. Pleskot, E. Plotnikova, D. Pluth, P. Podberezko, R. Poettgen, R. Poggi, L. Poggioli, D. Pohl, G. Polesello, A. Poley, A. Policicchio, R. Polifka, A. Polini, C. S. Pollard, V. Polychronakos, K. Pommès, D. Ponomarenko, L. Pontecorvo, B. G. Pope, G. A. Popeneciu, A. Poppleton, S. Pospisil, K. Potamianos, I. N. Potrap, C. J. Potter, G. Poulard, T. Poulsen, J. Poveda, M. E. Pozo Astigarraga, P. Pralavorio, A. Pranko, S. Prell, D. Price, L. E. Price, M. Primavera, S. Prince, N. Proklova, K. Prokofiev, F. Prokoshin, S. Protopopescu, J. Proudfoot, M. Przybycien, A. Puri, P. Puzo, J. Qian, G. Qin, Y. Qin, A. Quadt, M. Queitsch-Maitland, D. Quilty, S. Raddum, V. Radeka, V. Radescu, S. K. Radhakrishnan, P. Radloff, P. Rados, F. Ragusa, G. Rahal, J. A. Raine, S. Rajagopalan, C. Rangel-Smith, T. Rashid, S. Raspopov, M. G. Ratti, D. M. Rauch, F. Rauscher, S. Rave, I. Ravinovich, J. H. Rawling, M. Raymond, A. L. Read, N. P. Readioff, M. Reale, D. M. Rebuzzi, A. Redelbach, G. Redlinger, R. Reece, R. G. Reed, K. Reeves, L. Rehnisch, J. Reichert, A. Reiss, C. Rembser, H. Ren, M. Rescigno, S. Resconi, E. D. Resseguie, S. Rettie, E. Reynolds, O. L. Rezanova, P. Reznicek, R. Rezvani, R. Richter, S. Richter, E. Richter-Was, O. Ricken, M. Ridel, P. Rieck, C. J. Riegel, J. Rieger, O. Rifki, M. Rijssenbeek, A. Rimoldi, M. Rimoldi, L. Rinaldi, G. Ripellino, B. Ristić, E. Ritsch, I. Riu, F. Rizatdinova, E. Rizvi, C. Rizzi, R. T. Roberts, S. H. Robertson, A. Robichaud-Veronneau, D. Robinson, J. E. M. Robinson, A. Robson, E. Rocco, C. Roda, Y. Rodina, S. Rodriguez Bosca, A. Rodriguez Perez, D. Rodriguez Rodriguez, S. Roe, C. S. Rogan, O. Røhne, J. Roloff, A. Romaniouk, M. Romano, S. M. Romano Saez, E. Romero Adam, N. Rompotis, M. Ronzani, L. Roos, S. Rosati, K. Rosbach, P. Rose, N.-A. Rosien, E. Rossi, L. P. Rossi, J. H. N. Rosten, R. Rosten, M. Rotaru, I. Roth, J. Rothberg, D. Rousseau, A. Rozanov, Y. Rozen, X. Ruan, F. Rubbo, F. Rühr, A. Ruiz-Martinez, Z. Rurikova, N. A. Rusakovich, H. L. Russell, J. P. Rutherfoord, N. Ruthmann, Y. F. Ryabov, M. Rybar, G. Rybkin, S. Ryu, A. Ryzhov, G. F. Rzehorz, A. F. Saavedra, G. Sabato, S. Sacerdoti, H. F.-W. Sadrozinski, R. Sadykov, F. Safai Tehrani, P. Saha, M. Sahinsoy, M. Saimpert, M. Saito, T. Saito, H. Sakamoto, Y. Sakurai, G. Salamanna, J. E. Salazar Loyola, D. Salek, P. H. Sales De Bruin, D. Salihagic, A. Salnikov, J. Salt, D. Salvatore, F. Salvatore, A. Salvucci, A. Salzburger, D. Sammel, D. Sampsonidis, D. Sampsonidou, J. Sánchez, V. Sanchez Martinez, A. Sanchez Pineda, H. Sandaker, R. L. Sandbach, C. O. Sander, M. Sandhoff, C. Sandoval, D. P. C. Sankey, M. Sannino, A. Sansoni, C. Santoni, R. Santonico, H. Santos, I. Santoyo Castillo, A. Sapronov, J. G. Saraiva, B. Sarrazin, O. Sasaki, K. Sato, E. Sauvan, G. Savage, P. Savard, N. Savic, C. Sawyer, L. Sawyer, J. Saxon, C. Sbarra, A. Sbrizzi, T. Scanlon, D. A. Scannicchio, M. Scarcella, V. Scarfone, J. Schaarschmidt, P. Schacht, B. M. Schachtner, D. Schaefer, L. Schaefer, R. Schaefer, J. Schaeffer, S. Schaepe, S. Schaetzel, U. Schäfer, A. C. Schaffer, D. Schaile, R. D. Schamberger, V. Scharf, V. A. Schegelsky, D. Scheirich, M. Schernau, C. Schiavi, S. Schier, L. K. Schildgen, C. Schillo, M. Schioppa, S. Schlenker, K. R. Schmidt-Sommerfeld, K. Schmieden, C. Schmitt, S. Schmitt, S. Schmitz, U. Schnoor, L. Schoeffel, A. Schoening, B. D. Schoenrock, E. Schopf, M. Schott, J. F. P. Schouwenberg, J. Schovancova, S. Schramm, N. Schuh, A. Schulte, M. J. Schultens, H.-C. Schultz-Coulon, H. Schulz, M. Schumacher, B. A. Schumm, Ph. Schune, A. Schwartzman, T. A. Schwarz, H. Schweiger, Ph. Schwemling, R. Schwienhorst, J. Schwindling, A. Sciandra, G. Sciolla, F. Scuri, F. Scutti, J. Searcy, P. Seema, S. C. Seidel, A. Seiden, J. M. Seixas, G. Sekhniaidze, K. Sekhon, S. J. Sekula, N. Semprini-Cesari, S. Senkin, C. Serfon, L. Serin, L. Serkin, M. Sessa, R. Seuster, H. Severini, T. Sfiligoj, F. Sforza, A. Sfyrla, E. Shabalina, N. W. Shaikh, L. Y. Shan, R. Shang, J. T. Shank, M. Shapiro, P. B. Shatalov, K. Shaw, S. M. Shaw, A. Shcherbakova, C. Y. Shehu, Y. Shen, N. Sherafati, P. Sherwood, L. Shi, S. Shimizu, C. O. Shimmin, M. Shimojima, I. P. J. Shipsey, S. Shirabe, M. Shiyakova, J. Shlomi, A. Shmeleva, D. Shoaleh Saadi, M. J. Shochet, S. Shojaii, D. R. Shope, S. Shrestha, E. Shulga, M. A. Shupe, P. Sicho, A. M. Sickles, P. E. Sidebo, E. Sideras Haddad, O. Sidiropoulou, A. Sidoti, F. Siegert, Dj. Sijacki, J. Silva, S. B. Silverstein, V. Simak, Lj. Simic, S. Simion, E. Simioni, B. Simmons, M. Simon, P. Sinervo, N. B. Sinev, M. Sioli, G. Siragusa, I. Siral, S. Yu. Sivoklokov, J. Sjölin, M. B. Skinner, P. Skubic, M. Slater, T. Slavicek, M. Slawinska, K. Sliwa, R. Slovak, V. Smakhtin, B. H. Smart, J. Smiesko, N. Smirnov, S. Yu. Smirnov, Y. Smirnov, L. N. Smirnova, O. Smirnova, J. W. Smith, M. N. K. Smith, R. W. Smith, M. Smizanska, K. Smolek, A. A. Snesarev, I. M. Snyder, S. Snyder, R. Sobie, F. Socher, A. Soffer, D. A. Soh, G. Sokhrannyi, C. A. Solans Sanchez, M. Solar, E. Yu. Soldatov, U. Soldevila, A. A. Solodkov, A. Soloshenko, O. V. Solovyanov, V. Solovyev, P. Sommer, H. Son, A. Sopczak, D. Sosa, C. L. Sotiropoulou, R. Soualah, A. M. Soukharev, D. South, B. C. Sowden, S. Spagnolo, M. Spalla, M. Spangenberg, F. Spanò, D. Sperlich, F. Spettel, T. M. Spieker, R. Spighi, G. Spigo, L. A. Spiller, M. Spousta, R. D. St. Denis, A. Stabile, R. Stamen, S. Stamm, E. Stanecka, R. W. Stanek, C. Stanescu, M. M. Stanitzki, B. S. Stapf, S. Stapnes, E. A. Starchenko, G. H. Stark, J. Stark, S. H Stark, P. Staroba, P. Starovoitov, S. Stärz, R. Staszewski, P. Steinberg, B. Stelzer, H. J. Stelzer, O. Stelzer-Chilton, H. Stenzel, G. A. Stewart, M. C. Stockton, M. Stoebe, G. Stoicea, P. Stolte, S. Stonjek, A. R. Stradling, A. Straessner, M. E. Stramaglia, J. Strandberg, S. Strandberg, M. Strauss, P. Strizenec, R. Ströhmer, D. M. Strom, R. Stroynowski, A. Strubig, S. A. Stucci, B. Stugu, N. A. Styles, D. Su, J. Su, S. Suchek, Y. Sugaya, M. Suk, V. V. Sulin, D. M. S. Sultan, S. Sultansoy, T. Sumida, S. Sun, X. Sun, K. Suruliz, C. J. E. Suster, M. R. Sutton, S. Suzuki, M. Svatos, M. Swiatlowski, S. P. Swift, I. Sykora, T. Sykora, D. Ta, K. Tackmann, J. Taenzer, A. Taffard, R. Tafirout, N. Taiblum, H. Takai, R. Takashima, E. H. Takasugi, T. Takeshita, Y. Takubo, M. Talby, A. A. Talyshev, J. Tanaka, M. Tanaka, R. Tanaka, S. Tanaka, R. Tanioka, B. B. Tannenwald, S. Tapia Araya, S. Tapprogge, S. Tarem, G. F. Tartarelli, P. Tas, M. Tasevsky, T. Tashiro, E. Tassi, A. Tavares Delgado, Y. Tayalati, A. C. Taylor, G. N. Taylor, P. T. E. Taylor, W. Taylor, P. Teixeira-Dias, D. Temple, H. Ten Kate, P. K. Teng, J. J. Teoh, F. Tepel, S. Terada, K. Terashi, J. Terron, S. Terzo, M. Testa, R. J. Teuscher, T. Theveneaux-Pelzer, J. P. Thomas, J. Thomas-Wilsker, P. D. Thompson, A. S. Thompson, L. A. Thomsen, E. Thomson, M. J. Tibbetts, R. E. Ticse Torres, V. O. Tikhomirov, Yu. A. Tikhonov, S. Timoshenko, P. Tipton, S. Tisserant, K. Todome, S. Todorova-Nova, J. Tojo, S. Tokár, K. Tokushuku, E. Tolley, L. Tomlinson, M. Tomoto, L. Tompkins, K. Toms, B. Tong, P. Tornambe, E. Torrence, H. Torres, E. Torró Pastor, J. Toth, F. Touchard, D. R. Tovey, C. J. Treado, T. Trefzger, F. Tresoldi, A. Tricoli, I. M. Trigger, S. Trincaz-Duvoid, M. F. Tripiana, W. Trischuk, B. Trocmé, A. Trofymov, C. Troncon, M. Trottier-McDonald, M. Trovatelli, L. Truong, M. Trzebinski, A. Trzupek, K. W. Tsang, J. C.-L. Tseng, P. V. Tsiareshka, G. Tsipolitis, N. Tsirintanis, S. Tsiskaridze, V. Tsiskaridze, E. G. Tskhadadze, K. M. Tsui, I. I. Tsukerman, V. Tsulaia, S. Tsuno, D. Tsybychev, Y. Tu, A. Tudorache, V. Tudorache, T. T. Tulbure, A. N. Tuna, S. A. Tupputi, S. Turchikhin, D. Turgeman, I. Turk Cakir, R. Turra, P. M. Tuts, G. Ucchielli, I. Ueda, M. Ughetto, F. Ukegawa, G. Unal, A. Undrus, G. Unel, F. C. Ungaro, Y. Unno, C. Unverdorben, J. Urban, P. Urquijo, P. Urrejola, G. Usai, J. Usui, L. Vacavant, V. Vacek, B. Vachon, C. Valderanis, E. Valdes Santurio, S. Valentinetti, A. Valero, L. Valéry, S. Valkar, A. Vallier, J. A. Valls Ferrer, W. Van Den Wollenberg, H. van der Graaf, P. van Gemmeren, J. Van Nieuwkoop, I. van Vulpen, M. C. van Woerden, M. Vanadia, W. Vandelli, A. Vaniachine, P. Vankov, G. Vardanyan, R. Vari, E. W. Varnes, C. Varni, T. Varol, D. Varouchas, A. Vartapetian, K. E. Varvell, J. G. Vasquez, G. A. Vasquez, F. Vazeille, T. Vazquez Schroeder, J. Veatch, V. Veeraraghavan, L. M. Veloce, F. Veloso, S. Veneziano, A. Ventura, M. Venturi, N. Venturi, A. Venturini, V. Vercesi, M. Verducci, W. Verkerke, A. T. Vermeulen, J. C. Vermeulen, M. C. Vetterli, N. Viaux Maira, O. Viazlo, I. Vichou, T. Vickey, O. E. Vickey Boeriu, G. H. A. Viehhauser, S. Viel, L. Vigani, M. Villa, M. Villaplana Perez, E. Vilucchi, M. G. Vincter, V. B. Vinogradov, A. Vishwakarma, C. Vittori, I. Vivarelli, S. Vlachos, M. Vlasak, M. Vogel, P. Vokac, G. Volpi, H. von der Schmitt, E. von Toerne, V. Vorobel, K. Vorobev, M. Vos, R. Voss, J. H. Vossebeld, N. Vranjes, M. Vranjes Milosavljevic, V. Vrba, M. Vreeswijk, R. Vuillermet, I. Vukotic, P. Wagner, W. Wagner, J. Wagner-Kuhr, H. Wahlberg, S. Wahrmund, J. Wakabayashi, J. Walder, R. Walker, W. Walkowiak, V. Wallangen, C. Wang, C. Wang, F. Wang, H. Wang, H. Wang, J. Wang, J. Wang, Q. Wang, R. Wang, S. M. Wang, T. Wang, W. Wang, W. Wang, Z. Wang, C. Wanotayaroj, A. Warburton, C. P. Ward, D. R. Wardrope, A. Washbrook, P. M. Watkins, A. T. Watson, M. F. Watson, G. Watts, S. Watts, B. M. Waugh, A. F. Webb, S. Webb, M. S. Weber, S. W. Weber, S. A. Weber, J. S. Webster, A. R. Weidberg, B. Weinert, J. Weingarten, M. Weirich, C. Weiser, H. Weits, P. S. Wells, T. Wenaus, T. Wengler, S. Wenig, N. Wermes, M. D. Werner, P. Werner, M. Wessels, K. Whalen, N. L. Whallon, A. M. Wharton, A. S. White, A. White, M. J. White, R. White, D. Whiteson, B. W. Whitmore, F. J. Wickens, W. Wiedenmann, M. Wielers, C. Wiglesworth, L. A. M. Wiik-Fuchs, A. Wildauer, F. Wilk, H. G. Wilkens, H. H. Williams, S. Williams, C. Willis, S. Willocq, J. A. Wilson, I. Wingerter-Seez, E. Winkels, F. Winklmeier, O. J. Winston, B. T. Winter, M. Wittgen, M. Wobisch, T. M. H. Wolf, R. Wolff, M. W. Wolter, H. Wolters, V. W. S. Wong, S. D. Worm, B. K. Wosiek, J. Wotschack, K. W. Wozniak, M. Wu, S. L. Wu, X. Wu, Y. Wu, T. R. Wyatt, B. M. Wynne, S. Xella, Z. Xi, L. Xia, D. Xu, L. Xu, B. Yabsley, S. Yacoob, D. Yamaguchi, Y. Yamaguchi, A. Yamamoto, S. Yamamoto, T. Yamanaka, M. Yamatani, K. Yamauchi, Y. Yamazaki, Z. Yan, H. Yang, H. Yang, Y. Yang, Z. Yang, W.-M. Yao, Y. C. Yap, Y. Yasu, E. Yatsenko, K. H. Yau Wong, J. Ye, S. Ye, I. Yeletskikh, E. Yigitbasi, E. Yildirim, K. Yorita, K. Yoshihara, C. Young, C. J. S. Young, J. Yu, J. Yu, S. P. Y. Yuen, I. Yusuff, B. Zabinski, G. Zacharis, R. Zaidan, A. M. Zaitsev, N. Zakharchuk, J. Zalieckas, A. Zaman, S. Zambito, D. Zanzi, C. Zeitnitz, A. Zemla, J. C. Zeng, Q. Zeng, O. Zenin, T. Ženiš, D. Zerwas, D. Zhang, F. Zhang, G. Zhang, H. Zhang, J. Zhang, L. Zhang, L. Zhang, M. Zhang, P. Zhang, R. Zhang, R. Zhang, X. Zhang, Y. Zhang, Z. Zhang, X. Zhao, Y. Zhao, Z. Zhao, A. Zhemchugov, B. Zhou, C. Zhou, L. Zhou, M. Zhou, M. Zhou, N. Zhou, C. G. Zhu, H. Zhu, J. Zhu, Y. Zhu, X. Zhuang, K. Zhukov, A. Zibell, D. Zieminska, N. I. Zimine, C. Zimmermann, S. Zimmermann, Z. Zinonos, M. Zinser, M. Ziolkowski, L. Živković, G. Zobernig, A. Zoccoli, R. Zou, M. zur Nedden, L. Zwalinski, ATLAS Collaboration

**Affiliations:** 1 Department of Physics, University of Adelaide, Adelaide, Australia; 2 Physics Department, SUNY Albany, Albany, NY USA; 3 Department of Physics, University of Alberta, Edmonton, AB Canada; 4 Department of Physics, Ankara University, Ankara, Turkey; 5 Istanbul Aydin University, Istanbul, Turkey; 6 Division of Physics, TOBB University of Economics and Technology, Ankara, Turkey; 7 LAPP, CNRS/IN2P3 and Université Savoie Mont Blanc, Annecy-le-Vieux, France; 8 High Energy Physics Division, Argonne National Laboratory, Argonne, IL USA; 9 Department of Physics, University of Arizona, Tucson, AZ USA; 10 Department of Physics, The University of Texas at Arlington, Arlington, TX USA; 11 Physics Department, National and Kapodistrian University of Athens, Athens, Greece; 12 Physics Department, National Technical University of Athens, Zografou, Greece; 13 Department of Physics, The University of Texas at Austin, Austin, TX USA; 14 Institute of Physics, Azerbaijan Academy of Sciences, Baku, Azerbaijan; 15 Institut de Física d’Altes Energies (IFAE), The Barcelona Institute of Science and Technology, Barcelona, Spain; 16 Institute of Physics, University of Belgrade, Belgrade, Serbia; 17 Department for Physics and Technology, University of Bergen, Bergen, Norway; 18 Physics Division, Lawrence Berkeley National Laboratory, University of California, Berkeley, CA USA; 19 Department of Physics, Humboldt University, Berlin, Germany; 20 Albert Einstein Center for Fundamental Physics, Laboratory for High Energy Physics, University of Bern, Bern, Switzerland; 21 School of Physics and Astronomy, University of Birmingham, Birmingham, UK; 22 Department of Physics, Bogazici University, Istanbul, Turkey; 23 Department of Physics Engineering, Gaziantep University, Gaziantep, Turkey; 24 Faculty of Engineering and Natural Sciences, Istanbul Bilgi University, Istanbul, Turkey; 25 Faculty of Engineering and Natural Sciences, Bahcesehir University, Istanbul, Turkey; 26 Centro de Investigaciones, Universidad Antonio Narino, Bogotá, Colombia; 27 INFN Sezione di Bologna, Bologna, Italy; 28 Dipartimento di Fisica e Astronomia, Università di Bologna, Bologna, Italy; 29 Physikalisches Institut, University of Bonn, Bonn, Germany; 30 Department of Physics, Boston University, Boston, MA USA; 31 Department of Physics, Brandeis University, Waltham, MA USA; 32 Universidade Federal do Rio De Janeiro COPPE/EE/IF, Rio de Janeiro, Brazil; 33 Electrical Circuits Department, Federal University of Juiz de Fora (UFJF), Juiz de Fora, Brazil; 34 Federal University of Sao Joao del Rei (UFSJ), Sao Joao del Rei, Brazil; 35 Instituto de Fisica, Universidade de Sao Paulo, São Paulo, Brazil; 36 Physics Department, Brookhaven National Laboratory, Upton, NY USA; 37 Transilvania University of Brasov, Brasov, Romania; 38 Horia Hulubei National Institute of Physics and Nuclear Engineering, Bucharest, Romania; 39 Department of Physics, Alexandru Ioan Cuza University of Iasi, Iasi, Romania; 40 Physics Department, National Institute for Research and Development of Isotopic and Molecular Technologies, Cluj-Napoca, Romania; 41 University Politehnica Bucharest, Bucharest, Romania; 42 West University in Timisoara, Timisoara, Romania; 43 Departamento de Física, Universidad de Buenos Aires, Buenos Aires, Argentina; 44 Cavendish Laboratory, University of Cambridge, Cambridge, UK; 45 Department of Physics, Carleton University, Ottawa, ON Canada; 46 CERN, Geneva, Switzerland; 47 Enrico Fermi Institute, University of Chicago, Chicago, IL USA; 48 Departamento de Física, Pontificia Universidad Católica de Chile, Santiago, Chile; 49 Departamento de Física, Universidad Técnica Federico Santa María, Valparaiso, Chile; 50 Institute of High Energy Physics, Chinese Academy of Sciences, Beijing, China; 51 Department of Physics, Nanjing University, Nanjing, Jiangsu China; 52 Physics Department, Tsinghua University, Beijing, 100084 China; 53 Department of Modern Physics and State Key Laboratory of Particle Detection and Electronics, University of Science and Technology of China, Hefei, Anhui China; 54 School of Physics, Shandong University, Jinan, Shandong China; 55 Department of Physics and Astronomy, Key Laboratory for Particle Physics, Astrophysics and Cosmology, Ministry of Education, Shanghai Key Laboratory for Particle Physics and Cosmology, Shanghai Jiao Tong University, Shanghai (also at PKU-CHEP), Shanghai, China; 56 Université Clermont Auvergne, CNRS/IN2P3, LPC, Clermont-Ferrand, France; 57 Nevis Laboratory, Columbia University, Irvington, NY USA; 58 Niels Bohr Institute, University of Copenhagen, Copenhagen, Denmark; 59 INFN Gruppo Collegato di Cosenza, Laboratori Nazionali di Frascati, Frascati, Italy; 60 Dipartimento di Fisica, Università della Calabria, Rende, Italy; 61 Faculty of Physics and Applied Computer Science, AGH University of Science and Technology, Kraków, Poland; 62 Marian Smoluchowski Institute of Physics, Jagiellonian University, Kraków, Poland; 63 Institute of Nuclear Physics, Polish Academy of Sciences, Kraków, Poland; 64 Physics Department, Southern Methodist University, Dallas, TX USA; 65 Physics Department, University of Texas at Dallas, Richardson, TX USA; 66 DESY, Hamburg and Zeuthen, Germany; 67 Lehrstuhl für Experimentelle Physik IV, Technische Universität Dortmund, Dortmund, Germany; 68 Institut für Kern- und Teilchenphysik, Technische Universität Dresden, Dresden, Germany; 69 Department of Physics, Duke University, Durham, NC USA; 70 SUPA-School of Physics and Astronomy, University of Edinburgh, Edinburgh, UK; 71 INFN Laboratori Nazionali di Frascati, Frascati, Italy; 72 Fakultät für Mathematik und Physik, Albert-Ludwigs-Universität, Freiburg, Germany; 73 Departement de Physique Nucleaire et Corpusculaire, Université de Genève, Geneva, Switzerland; 74 INFN Sezione di Genova, Genoa, Italy; 75 Dipartimento di Fisica, Università di Genova, Genoa, Italy; 76 E. Andronikashvili Institute of Physics, Iv. Javakhishvili Tbilisi State University, Tbilisi, Georgia; 77 High Energy Physics Institute, Tbilisi State University, Tbilisi, Georgia; 78 II Physikalisches Institut, Justus-Liebig-Universität Giessen, Giessen, Germany; 79 SUPA-School of Physics and Astronomy, University of Glasgow, Glasgow, UK; 80 II Physikalisches Institut, Georg-August-Universität, Göttingen, Germany; 81 Laboratoire de Physique Subatomique et de Cosmologie, Université Grenoble-Alpes, CNRS/IN2P3, Grenoble, France; 82 Laboratory for Particle Physics and Cosmology, Harvard University, Cambridge, MA USA; 83 Kirchhoff-Institut für Physik, Ruprecht-Karls-Universität Heidelberg, Heidelberg, Germany; 84 Physikalisches Institut, Ruprecht-Karls-Universität Heidelberg, Heidelberg, Germany; 85 ZITI Institut für technische Informatik, Ruprecht-Karls-Universität Heidelberg, Mannheim, Germany; 86 Faculty of Applied Information Science, Hiroshima Institute of Technology, Hiroshima, Japan; 87 Department of Physics, The Chinese University of Hong Kong, Shatin, NT Hong Kong; 88 Department of Physics, The University of Hong Kong, Hong Kong, China; 89 Department of Physics, Institute for Advanced Study, The Hong Kong University of Science and Technology, Clear Water Bay, Kowloon, Hong Kong, China; 90 Department of Physics, National Tsing Hua University, Taiwan, Taiwan; 91 Department of Physics, Indiana University, Bloomington, IN USA; 92 Institut für Astro- und Teilchenphysik, Leopold-Franzens-Universität, Innsbruck, Austria; 93 University of Iowa, Iowa City, IA USA; 94 Department of Physics and Astronomy, Iowa State University, Ames, IA USA; 95 Joint Institute for Nuclear Research, JINR Dubna, Dubna, Russia; 96 KEK, High Energy Accelerator Research Organization, Tsukuba, Japan; 97 Graduate School of Science, Kobe University, Kobe, Japan; 98 Faculty of Science, Kyoto University, Kyoto, Japan; 99 Kyoto University of Education, Kyoto, Japan; 100 Research Center for Advanced Particle Physics and Department of Physics, Kyushu University, Fukuoka, Japan; 101 Instituto de Física La Plata, Universidad Nacional de La Plata and CONICET, La Plata, Argentina; 102 Physics Department, Lancaster University, Lancaster, UK; 103 INFN Sezione di Lecce, Lecce, Italy; 104 Dipartimento di Matematica e Fisica, Università del Salento, Lecce, Italy; 105 Oliver Lodge Laboratory, University of Liverpool, Liverpool, UK; 106 Department of Experimental Particle Physics, Jožef Stefan Institute and Department of Physics, University of Ljubljana, Ljubljana, Slovenia; 107 School of Physics and Astronomy, Queen Mary University of London, London, UK; 108 Department of Physics, Royal Holloway University of London, Surrey, UK; 109 Department of Physics and Astronomy, University College London, London, UK; 110 Louisiana Tech University, Ruston, LA USA; 111 Laboratoire de Physique Nucléaire et de Hautes Energies, UPMC and Université Paris-Diderot and CNRS/IN2P3, Paris, France; 112 Fysiska institutionen, Lunds universitet, Lund, Sweden; 113 Departamento de Fisica Teorica C-15, Universidad Autonoma de Madrid, Madrid, Spain; 114 Institut für Physik, Universität Mainz, Mainz, Germany; 115 School of Physics and Astronomy, University of Manchester, Manchester, UK; 116 CPPM, Aix-Marseille Université and CNRS/IN2P3, Marseille, France; 117 Department of Physics, University of Massachusetts, Amherst, MA USA; 118 Department of Physics, McGill University, Montreal, QC Canada; 119 School of Physics, University of Melbourne, Victoria, Australia; 120 Department of Physics, The University of Michigan, Ann Arbor, MI USA; 121 Department of Physics and Astronomy, Michigan State University, East Lansing, MI USA; 122 INFN Sezione di Milano, Milan, Italy; 123 Dipartimento di Fisica, Università di Milano, Milan, Italy; 124 B.I. Stepanov Institute of Physics, National Academy of Sciences of Belarus, Minsk, Republic of Belarus; 125 Research Institute for Nuclear Problems of Byelorussian State University, Minsk, Republic of Belarus; 126 Group of Particle Physics, University of Montreal, Montreal, QC Canada; 127 P.N. Lebedev Physical Institute of the Russian Academy of Sciences, Moscow, Russia; 128 Institute for Theoretical and Experimental Physics (ITEP), Moscow, Russia; 129 National Research Nuclear University MEPhI, Moscow, Russia; 130 D.V. Skobeltsyn Institute of Nuclear Physics, M.V. Lomonosov Moscow State University, Moscow, Russia; 131 Fakultät für Physik, Ludwig-Maximilians-Universität München, Munich, Germany; 132 Max-Planck-Institut für Physik (Werner-Heisenberg-Institut), Munich, Germany; 133 Nagasaki Institute of Applied Science, Nagasaki, Japan; 134 Graduate School of Science and Kobayashi-Maskawa Institute, Nagoya University, Nagoya, Japan; 135 INFN Sezione di Napoli, Naples, Italy; 136 Dipartimento di Fisica, Università di Napoli, Naples, Italy; 137 Department of Physics and Astronomy, University of New Mexico, Albuquerque, NM USA; 138 Institute for Mathematics, Astrophysics and Particle Physics, Radboud University Nijmegen/Nikhef, Nijmegen, The Netherlands; 139 Nikhef National Institute for Subatomic Physics, University of Amsterdam, Amsterdam, The Netherlands; 140 Department of Physics, Northern Illinois University, DeKalb, IL USA; 141 Budker Institute of Nuclear Physics, SB RAS, Novosibirsk, Russia; 142 Department of Physics, New York University, New York, NY USA; 143 Ohio State University, Columbus, OH USA; 144 Faculty of Science, Okayama University, Okayama, Japan; 145 Homer L. Dodge Department of Physics and Astronomy, University of Oklahoma, Norman, OK USA; 146 Department of Physics, Oklahoma State University, Stillwater, OK USA; 147 Palacký University, RCPTM, Olomouc, Czech Republic; 148 Center for High Energy Physics, University of Oregon, Eugene, OR USA; 149 LAL, Univ. Paris-Sud, CNRS/IN2P3, Université Paris-Saclay, Orsay, France; 150 Graduate School of Science, Osaka University, Osaka, Japan; 151 Department of Physics, University of Oslo, Oslo, Norway; 152 Department of Physics, Oxford University, Oxford, UK; 153 INFN Sezione di Pavia, Pavia, Italy; 154 Dipartimento di Fisica, Università di Pavia, Pavia, Italy; 155 Department of Physics, University of Pennsylvania, Philadelphia, PA USA; 156 National Research Centre “Kurchatov Institute” B.P. Konstantinov Petersburg Nuclear Physics Institute, St. Petersburg, Russia; 157 INFN Sezione di Pisa, Pisa, Italy; 158 Dipartimento di Fisica E. Fermi, Università di Pisa, Pisa, Italy; 159 Department of Physics and Astronomy, University of Pittsburgh, Pittsburgh, PA USA; 160 Laboratório de Instrumentação e Física Experimental de Partículas-LIP, Lisbon, Portugal; 161 Faculdade de Ciências, Universidade de Lisboa, Lisbon, Portugal; 162 Department of Physics, University of Coimbra, Coimbra, Portugal; 163 Centro de Física Nuclear da Universidade de Lisboa, Lisbon, Portugal; 164 Departamento de Fisica, Universidade do Minho, Braga, Portugal; 165 Departamento de Fisica Teorica y del Cosmos and CAFPE, Universidad de Granada, Granada, Spain; 166 Dep Fisica and CEFITEC of Faculdade de Ciencias e Tecnologia, Universidade Nova de Lisboa, Caparica, Portugal; 167 Institute of Physics, Academy of Sciences of the Czech Republic, Prague, Czech Republic; 168 Czech Technical University in Prague, Prague, Czech Republic; 169 Faculty of Mathematics and Physics, Charles University, Prague, Czech Republic; 170 State Research Center Institute for High Energy Physics (Protvino), NRC KI, Protvino, Russia; 171 Particle Physics Department, Rutherford Appleton Laboratory, Didcot, UK; 172 INFN Sezione di Roma, Rome, Italy; 173 Dipartimento di Fisica, Sapienza Università di Roma, Rome, Italy; 174 INFN Sezione di Roma Tor Vergata, Rome, Italy; 175 Dipartimento di Fisica, Università di Roma Tor Vergata, Rome, Italy; 176 INFN Sezione di Roma Tre, Rome, Italy; 177 Dipartimento di Matematica e Fisica, Università Roma Tre, Rome, Italy; 178 Faculté des Sciences Ain Chock, Réseau Universitaire de Physique des Hautes Energies-Université Hassan II, Casablanca, Morocco; 179 Centre National de l’Energie des Sciences Techniques Nucleaires, Rabat, Morocco; 180 Faculté des Sciences Semlalia, Université Cadi Ayyad, LPHEA-Marrakech, Marrakech, Morocco; 181 Faculté des Sciences, Université Mohamed Premier and LPTPM, Oujda, Morocco; 182 Faculté des Sciences, Université Mohammed V, Rabat, Morocco; 183 DSM/IRFU (Institut de Recherches sur les Lois Fondamentales de l’Univers), CEA Saclay (Commissariat à l’Energie Atomique et aux Energies Alternatives), Gif-sur-Yvette, France; 184 Santa Cruz Institute for Particle Physics, University of California Santa Cruz, Santa Cruz, CA USA; 185 Department of Physics, University of Washington, Seattle, WA USA; 186 Department of Physics and Astronomy, University of Sheffield, Sheffield, UK; 187 Department of Physics, Shinshu University, Nagano, Japan; 188 Department Physik, Universität Siegen, Siegen, Germany; 189 Department of Physics, Simon Fraser University, Burnaby, BC Canada; 190 SLAC National Accelerator Laboratory, Stanford, CA USA; 191 Faculty of Mathematics, Physics and Informatics, Comenius University, Bratislava, Slovak Republic; 192 Department of Subnuclear Physics, Institute of Experimental Physics of the Slovak Academy of Sciences, Kosice, Slovak Republic; 193 Department of Physics, University of Cape Town, Cape Town, South Africa; 194 Department of Physics, University of Johannesburg, Johannesburg, South Africa; 195 School of Physics, University of the Witwatersrand, Johannesburg, South Africa; 196 Department of Physics, Stockholm University, Stockholm, Sweden; 197 The Oskar Klein Centre, Stockholm, Sweden; 198 Physics Department, Royal Institute of Technology, Stockholm, Sweden; 199 Departments of Physics and Astronomy and Chemistry, Stony Brook University, Stony Brook, NY USA; 200 Department of Physics and Astronomy, University of Sussex, Brighton, UK; 201 School of Physics, University of Sydney, Sydney, Australia; 202 Institute of Physics, Academia Sinica, Taipei, Taiwan; 203 Department of Physics, Technion: Israel Institute of Technology, Haifa, Israel; 204 Raymond and Beverly Sackler School of Physics and Astronomy, Tel Aviv University, Tel Aviv, Israel; 205 Department of Physics, Aristotle University of Thessaloniki, Thessaloníki, Greece; 206 International Center for Elementary Particle Physics and Department of Physics, The University of Tokyo, Tokyo, Japan; 207 Graduate School of Science and Technology, Tokyo Metropolitan University, Tokyo, Japan; 208 Department of Physics, Tokyo Institute of Technology, Tokyo, Japan; 209 Tomsk State University, Tomsk, Russia; 210 Department of Physics, University of Toronto, Toronto, ON Canada; 211 INFN-TIFPA, Trento, Italy; 212 University of Trento, Trento, Italy; 213 TRIUMF, Vancouver, BC Canada; 214 Department of Physics and Astronomy, York University, Toronto, ON Canada; 215 Faculty of Pure and Applied Sciences, and Center for Integrated Research in Fundamental Science and Engineering, University of Tsukuba, Tsukuba, Japan; 216 Department of Physics and Astronomy, Tufts University, Medford, MA USA; 217 Department of Physics and Astronomy, University of California Irvine, Irvine, CA USA; 218 INFN Gruppo Collegato di Udine, Sezione di Trieste, Udine, Italy; 219 ICTP, Trieste, Italy; 220 Dipartimento di Chimica, Fisica e Ambiente, Università di Udine, Udine, Italy; 221 Department of Physics and Astronomy, University of Uppsala, Uppsala, Sweden; 222 Department of Physics, University of Illinois, Urbana, IL USA; 223 Instituto de Fisica Corpuscular (IFIC), Centro Mixto Universidad de Valencia - CSIC, Valencia, Spain; 224 Department of Physics, University of British Columbia, Vancouver, BC Canada; 225 Department of Physics and Astronomy, University of Victoria, Victoria, BC Canada; 226 Department of Physics, University of Warwick, Coventry, UK; 227 Waseda University, Tokyo, Japan; 228 Department of Particle Physics, The Weizmann Institute of Science, Rehovot, Israel; 229 Department of Physics, University of Wisconsin, Madison, WI USA; 230 Fakultät für Physik und Astronomie, Julius-Maximilians-Universität, Würzburg, Germany; 231 Fakultät für Mathematik und Naturwissenschaften, Fachgruppe Physik, Bergische Universität Wuppertal, Wuppertal, Germany; 232 Department of Physics, Yale University, New Haven, CT USA; 233 Yerevan Physics Institute, Yerevan, Armenia; 234 1211, Geneva 23, Switzerland; 235 Centre de Calcul de l’Institut National de Physique Nucléaire et de Physique des Particules (IN2P3), Villeurbanne, France; 236 Academia Sinica Grid Computing, Institute of Physics, Academia Sinica, Taipei, Taiwan; 237 CERN, 1211 Geneva 23, Switzerland

## Abstract

Results of a search for physics beyond the Standard Model in events containing an energetic photon and large missing transverse momentum with the ATLAS detector at the Large Hadron Collider are reported. As the number of events observed in data, corresponding to an integrated luminosity of 36.1 fb$^{-1}$  of proton–proton collisions at a centre-of-mass energy of $13~\mathrm{TeV}$, is in agreement with the Standard Model expectations, model-independent limits are set on the fiducial cross section for the production of events in this final state. Exclusion limits are also placed in models where dark-matter candidates are pair-produced. For dark-matter production via an axial-vector or a vector mediator in the *s*-channel, this search excludes mediator masses below 750–$1200~\mathrm{GeV}$ for dark-matter candidate masses below 230–$480~\mathrm{GeV}$ at 95% confidence level, depending on the couplings. In an effective theory of dark-matter production, the limits restrict the value of the suppression scale $M_{*}$ to be above $790~\mathrm{GeV}$ at 95% confidence level. A limit is also reported on the production of a high-mass scalar resonance by processes beyond the Standard Model, in which the resonance decays to $Z\gamma$ and the *Z* boson subsequently decays into neutrinos.

## Introduction

Multiple theories of physics beyond the Standard Model (BSM) predict a high production rate of events containing a photon with a high transverse energy ($E_{\mathrm {T}}^{\gamma }$) and large missing transverse momentum (${\varvec{E}}_{\text {T}}^{\text {miss}}$, with magnitude $E_{\text {T}}^{\text {miss}}$) referred to as $\gamma +E_{\text {T}}^{\text {miss}}$ events, in *pp* collisions. The search for energetic $\gamma +E_{\text {T}}^{\text {miss}}$ events, whose rates have a low expected contribution from Standard Model (SM) processes, can thus provide sensitivity to new physics models [1–5], also related to dark matter (DM). Although the existence of DM is well established [6], its nature is yet unknown. One candidate is a weakly interacting massive particle (WIMP, also denoted by $\chi$) that interacts with SM particles with a strength similar to the weak interaction. If WIMPs interact with quarks via a mediator particle, pairs of WIMPs are produced in *pp* collisions at sufficiently high energy. The $\chi \bar{\chi }$ pair is invisible to the detector, but the radiation of an initial-state photon in $q\bar{q}\rightarrow \chi \bar{\chi }$ interactions [7] can produce detectable $\gamma +E_{\text {T}}^{\text {miss}}$ events.

**Fig. 1:**
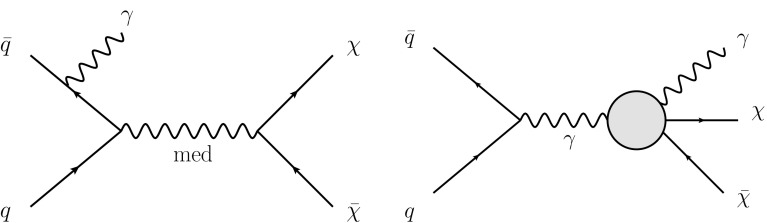
Pair production of dark-matter particles ($\chi \bar{\chi }$) in association with a photon via an explicit *s*-channel mediator (*left*), or via an effective $\gamma \gamma \chi \bar{\chi }$ vertex (*right*)

Effective field theories (EFT) with various forms of interaction between the WIMPs and the SM particles are a powerful model-independent approach for the interpretation of DM production in *pp* collisions [7]. However, the typical momentum transfer in *pp* collisions at the LHC can often exceed the cut-off scale below which the EFT approximation is valid. Simplified models that involve the explicit production of the intermediate state, as shown in Fig. 1 (left), avoid this limitation. This paper focuses on simplified models assuming Dirac-fermion DM candidates produced via an *s*-channel mediator with vector or axial-vector interactions [8–10]. There are five free parameters in this model: the WIMP mass $m_\chi$, the mediator mass $m_{\text {med}}$, the width of the mediator $\Gamma _{\text {med}}$, the coupling $g_q$ of the mediator to quarks, and the coupling $g_{\chi }$ of the mediator to the dark-matter particle. In the limit of a large mediator mass, these simplified models map onto the EFT operators, with the suppression scale^1^ $M_{*}$ linked to $m_{\text {med}}$ by the relation $M_{*}=m_{\text {med}}/\sqrt{g_qg_\chi }$ [11].

The paper also considers a specific dimension-7 EFT operator with direct couplings between DM and electroweak (EW) bosons, for which there is neither a corresponding simplified model nor a simplified model yielding similar kinematic distributions implemented in an event generator [10, 12]. The process describing a contact interaction of type $\gamma \gamma \chi \bar{\chi }$ is shown in Fig. 1 (right). In this model, DM production proceeds via $q \bar{q} \rightarrow \gamma \rightarrow \gamma \chi \bar{\chi }$, generating an energetic photon without requiring initial-state radiation. There are four free parameters in this model: the EW coupling strengths $k_1$ and $k_2$ (which respectively control the strength of the coupling to the SM U(1) and SU(2) gauge sectors), $m_{\chi }$, and the suppression scale $M_{*}$.

Many BSM models [13, 14] introduce new bosons through either an extension of the Higgs sector or additional gauge fields. In some of those, the bosons are predicted to decay into electroweak gauge bosons: the analysis presented here also searches for such a resonance decaying into $Z\gamma$, which would lead to an excess of energetic $\gamma$+$E_{\text {T}}^{\text {miss}}$ events when the *Z* boson subsequently decays to neutrinos.

The ATLAS and CMS collaborations have reported limits in various models based on searches for an excess of $\gamma +E_{\text {T}}^{\text {miss}}$ events using *pp* collisions at centre-of-mass energies of $\sqrt{s}=7$ and $8 ~\mathrm{TeV}$ (LHC Run 1) and with the first LHC Run-2 data collected in 2015 at a centre-of-mass energy of $13~\mathrm{TeV}$ [15–19]. A $\chi \bar{\chi }$ pair can also be produced in association with other objects leading to different *X*+$E_{\text {T}}^{\text {miss}}$ signatures, where *X* can be a jet, a *W* boson, a *Z* boson or a Higgs boson. DM searches are hence performed in a variety of complementary final states [20–24]. The $\gamma +E_{\text {T}}^{\text {miss}}$ final state has the advantage of a clean signature providing a good complementarity with respect to the other *X*+$E_{\text {T}}^{\text {miss}}$ processes. Moreover it also offers the unique possibility to probe for DM models in which the photon does not come from initial-state radiation. This paper reports the results of a search for dark matter and for a BSM $Z\gamma$ resonance in $\gamma +E_{\text {T}}^{\text {miss}}$ events in *pp* collisions at a centre-of-mass energy of $\sqrt{s}=13~\mathrm{TeV}$ using the Run-2 data collected in 2015 and 2016, corresponding to an integrated luminosity of 36.1 fb$^{-1}$. As described in Sect. 5, this search follows a strategy similar to that implemented in Ref. [17], but with multiple signal regions optimised to take advantage of the tenfold increase in integrated luminosity.

The paper is organised as follows. A brief description of the ATLAS detector is given in Sect. 2. The signal and background Monte Carlo (MC) simulation samples used are described in Sect. 3. The reconstruction of physics objects is explained in Sect. 4, and the event selection is described in Sect. 5. Estimation of the SM backgrounds is outlined in Sect. 6. The results are described in Sect. 7 and the systematic uncertainties are given in Sect. 8. The interpretation of results in terms of models of pair production of dark-matter candidates and of BSM production of a high-mass $Z\gamma$ resonance is described in Sect. 9. A summary is given in Sect. 10.

## The ATLAS detector

The ATLAS detector [25] is a multipurpose particle physics apparatus with a forward–backward symmetric cylindrical geometry and near 4$\pi$ coverage in solid angle.^2^ The inner tracking detector (ID), covering the pseudorapidity range $|\eta |<$ 2.5, consists of a silicon pixel detector including the insertable B-layer [26, 27], which was added around a new, smaller-radius beam-pipe before the start of Run 2; a silicon microstrip detector; and, for $|\eta |<$ 2.0, a straw-tube transition radiation tracker (TRT). The ID is surrounded by a thin superconducting solenoid which provides a 2 T magnetic field. A high-granularity lead/liquid-argon sampling electromagnetic calorimeter (EM) covers the region $|\eta |<$ 3.2. It is segmented longitudinally in shower depth. The first layer has a high granularity in the $\eta$ direction in order to provide an efficient discrimination between single-photon showers and two overlapping photons originating from a $\pi ^0$ decay. The second layer is where most of the energy, deposited in the calorimeter by electron- or photon-initiated electromagnetic showers, is collected. Significant energy deposits can be left in the third layer by very high energy showers; this layer can also be used to correct for energy leakage beyond the electromagnetic calorimeter. A steel/scintillator-tile hadronic calorimeter covers the range $|\eta |<$ 1.7, while the liquid-argon technology with either copper or tungsten as the absorber material is used for the hadronic calorimeters in the end-cap region 1.5 $<|\eta |<$ 3.2 and for electromagnetic and hadronic measurements in the forward region up to $|\eta |=$ 4.9. A muon spectrometer (MS) surrounds the calorimeters. It consists of three large air-core superconducting toroidal magnet systems, precision tracking chambers providing accurate muon tracking out to $|\eta |$ = 2.7, and fast detectors for triggering in the region $|\eta |<$ 2.4. A two-level trigger system is used to select events for offline analysis [28].

## Monte Carlo simulation samples

Several simulated MC samples are used to estimate the signal acceptance, the detector efficiency and various SM background contributions. For all the DM samples considered here, the values of the free parameters were chosen following the recommendations given in Ref. [10].

Samples of DM production in simplified models are generated via an *s*-channel mediator with axial-vector interactions. The program MG5_aMC@NLO v2.4.3 [29] is used in conjunction with $\textsc {Pythia}$ v8.212 [30] with the parameter values set according to the ATLAS tune A14 [31]. The parton distribution function (PDF) set used is NNPDF3.0 at next-to-leading order (NLO) [32] with $\alpha _{\mathrm {s}} = 0.118.$ The $g_q$ coupling is set to be universal in quark flavour and equal to 0.25, the $g_{\chi }$ coupling is set to 1.0, and $\Gamma _{\text {med}}$ is computed as the minimum width allowed given the couplings and masses. As shown in Ref. [10], $\Gamma _{\text {med}}/m_{\text {med}}$ varies between 2 and 6% for the values probed here. Different choices of the couplings and a model with a vector mediator are also considered, as described in Sect. 9. The generation was updated with respect to the 2015 data analysis [17] by using the DMsimp [33] implementation of the model at NLO. Events are generated with parameters spanning a grid of points in the $m_{\chi }$–$m_{\text {med}}$ plane.

For DM samples corresponding to an EFT model involving dimension-7 operators with a contact interaction of type $\gamma \gamma \chi \bar{\chi }$, the parameters which only influence the cross section are set to $k_1=k_2=1.0$ and $M_*=3.0~\mathrm{TeV}$ [10]. A scan over a range of values of $m_{\chi }$ is performed. Events are generated with MG5_aMC@NLO v2.2.3 and the PDF set NNPDF3.0 at leading order (LO) with $\alpha _s=0.130,$ in conjunction with $\textsc {Pythia}$ v8.186, using the ATLAS tune A14.

For DM signal generation in both the simplified and EFT models, a photon with at least $E_{\mathrm {T}}^{\gamma }= 130~\mathrm{GeV}$ is required at the matrix-element level in MG5_aMC@NLO.

The samples used in the search for a BSM high-mass scalar resonance decaying to $Z\gamma$ are generated using Powheg-Box v1 [34], with the CT10 PDF set [35] and $\textsc {Pythia}$ v8.210 for the showering with the AZNLO tune [36] based on the CTEQ6L1 PDF set [37]. The simulated heavy scalar resonance *X* of very narrow width (4 MeV), with masses in the range 2 to $5~\mathrm{TeV}$, is produced through gluon–gluon fusion and then assumed to decay exclusively to $Z\gamma$.

For all the signal samples described above, the EvtGen v1.2.0 program [38] is used for properties of the bottom and charm hadron decays.

For $W\gamma$ and $Z\gamma$ backgrounds, events containing a charged lepton (*e*, $\mu$ or $\tau$) and a neutrino, a pair of neutrinos ($\nu \nu$) or a pair of charged leptons ($\ell \ell$) together with a photon and associated jets are simulated using the $\textsc {Sherpa}$ v2.1.1 event generator [39]. The matrix elements including all diagrams with three electroweak couplings are calculated with up to three partons at LO and merged with $\textsc {Sherpa}$ parton showers [40] using the ME+PS@LO prescription [41]. The CT10 PDF set is used in conjunction with a dedicated parton shower tuning developed by the $\textsc {Sherpa}$ authors. For *Z* events with the *Z* boson decaying to a $\ell \ell$ pair a requirement on the dilepton invariant mass of $m_{\ell \ell } > 10 ~\mathrm{GeV}$ is applied at event generator level.

Events containing a photon with associated jets are also simulated using $\textsc {Sherpa}$ v2.1.1 [39], generated in several bins of $E_{\mathrm {T}}^{\gamma }$ with lower edges ranging from $35~\mathrm{GeV}$ to $4~\mathrm{TeV}$. The matrix elements are calculated at LO with up to three or four partons and merged with $\textsc {Sherpa}$ parton showers using the ME+PS@LO prescription. The CT10 PDF set is used in conjunction with the dedicated parton shower tuning.

For *W* / *Z*+jets backgrounds, events containing *W* or *Z* bosons with associated jets are simulated using $\textsc {Sherpa}$ v2.2.0. The matrix elements are calculated for up to four partons at LO and two partons at NLO using the Comix [42] and OpenLoops [43] matrix-element generators and merged with $\textsc {Sherpa}$ parton showers using the ME+PS@NLO prescription [44]. The NNPDF3.0 PDF set at next-to-next-to-leading order (NNLO) is used. As in the case of the $\gamma$+jets samples, the dedicated parton shower tuning is used. The *W* / *Z*+jets events are normalised to the NNLO inclusive cross sections [45].

Table 1 summarises the choices made in the generation of MC samples used in the analysis.

**Table 1:** Details of the generation of the signal samples and of the SM background processes considered in the analysis

Process	Event generators used	PDF sets	Order	Requirements
DMsimp model	MG5_aMC@NLO v2.4.3 $+$ $\textsc {Pythia}$ v8.212	NNPDF30_nlo_as_0118	NLO	$E_{\mathrm {T}}^{\gamma }> 130~\mathrm{GeV}$
EFT model	MG5_aMC@NLO v2.2.3 $+$ $\textsc {Pythia}$ v8.186	NNPDF30_lo_as_0130	LO	$E_{\mathrm {T}}^{\gamma }> 130~\mathrm{GeV}$
BSM resonance	Powheg-Box v1 $+$ $\textsc {Pythia}$ v8.210	CT10	NLO	–
$W/Z\gamma$	$\textsc {Sherpa}$ v2.1.1	CT10	LO	For *Z*: $m_{\ell \ell } > 10 ~\mathrm{GeV}$
$\gamma + {\mathrm {jets}}$	$\textsc {Sherpa}$ v2.1.1	CT10	LO	–
*W* / *Z*+jets	$\textsc {Sherpa}$ v2.2.0	NNPDF30_nnlo	LO/NLO	–

Multiple *pp* interactions in the same or neighbouring bunch crossings (referred to as pile-up) superimposed on the hard physics process are simulated with the minimum-bias processes of $\textsc {Pythia}$ v8.186 using the A2 tune [46] and the MSTW2008LO PDF set [47]. Simulated events are reweighted so that the distribution of the expected number of collisions per bunch crossing, $\langle \mu \rangle$, matches the one observed in data, which has a mean value of 13.7 (24.2) in 2015 (2016) data.

All generated event samples are processed with a full ATLAS detector simulation [48] based on Geant4 [49]. The simulated events are reconstructed and analysed with the same analysis chain as used for the data, utilising the same trigger and event selection criteria discussed in Sect. 5.

## Event reconstruction

Photons are reconstructed from clusters of energy deposits in the electromagnetic calorimeter measured in projective towers. Clusters without matching tracks are classified as unconverted photon candidates. A photon candidate containing clusters that can be matched to tracks is considered as a converted photon candidate [50]. The photon energy is corrected by applying the energy scales measured with $Z \rightarrow e^+ e^-$ decays [51]. The trajectory of the photon is reconstructed using the longitudinal (shower depth) segmentation of the calorimeters and a constraint from the average collision point of the proton beams. For converted photons, the position of the conversion vertex is also used if tracks from the conversion have hits in the silicon detectors. Identification requirements are applied in order to reduce the contamination from $\pi ^0$ or other neutral hadrons decaying to two photons. The photon identification is based on the profile of the energy deposits in the first and second layers of the electromagnetic calorimeter. Candidate photons are required to have $E_{\mathrm {T}}^{\gamma }> 10~\mathrm{GeV}$, to satisfy the “loose” identification criteria defined in Ref. [52] and to be within $|\eta | < 2.37$. Photons used in the event selection must additionally satisfy the “tight” identification criteria [52], have $|\eta |<1.37$ or $1.52<|\eta |<2.37$ and be isolated by requiring the energy in the calorimeters in a cone of size $\Delta R = \sqrt{(\Delta \eta )^2 + (\Delta \phi )^2} = 0.4$ around the cluster barycentre, excluding the energy associated with the photon cluster, to be less than $2.45~\mathrm{GeV}+ 0.022\times E_{\mathrm {T}}^{\gamma }$. This cone energy is corrected for the leakage of the photon energy from the central core and for the effects of pile-up [51]. In addition, the scalar sum of the $p_{\text {T}}$ of non-conversion tracks in a cone of size $\Delta R = 0.2$ around the cluster barycentre is required to be less than $0.05\times E_{\mathrm {T}}^{\gamma }$.

Electrons are reconstructed from clusters in the electromagnetic calorimeter which are matched to a track in the ID. The criteria for their identification, and the calibration steps, are similar to those used for photons. Electron candidates must fulfil the “medium” identification requirement of Ref. [51]. Muons are identified either as a combined track in the MS and ID systems, or as an ID track that, once extrapolated to the MS, is associated with at least one track segment in the MS. Extrapolated muons are also considered; they are reconstructed from an MS track which is required to be compatible with originating from the nominal interaction point. Muon candidates must pass the “medium” identification requirement [53]. The significance of the transverse impact parameter, defined as the transverse impact parameter $d_0$ divided by its estimated uncertainty, $\sigma _{d_0}$, of tracks with respect to the beam line is required to satisfy $|d_0|/\sigma _{d_0}<$ 5.0 for electrons and $|d_0|/\sigma _{d_0}<$ 3.0 for muons. The longitudinal impact parameter $z_0$ must satisfy $|z_0 \sin \theta | <0.5$ mm for both the electrons and muons. Electrons are required to have $p_{\text {T}} > 7~\mathrm{GeV}$ and $|\eta | < 2.47$, while muons are required to have $p_{\text {T}} > 6~\mathrm{GeV}$ and $|\eta | < 2.7$.

Jets are reconstructed with the anti-$$k_{t}$$ algorithm [54] with a radius parameter $$R = $$ 0.4 from clusters of energy deposits at the electromagnetic scale in the calorimeters [55]. A correction used to calibrate the jet energy to the scale of its constituent particles [56, 57] is then applied. Jets are also corrected for contributions from pile-up interactions and a residual correction derived in situ is applied as the last step to jets reconstructed in data [56]. Candidate jets are required to have $$p_{\text {T}} > 20~\mathrm{GeV}$$. In order to suppress pile-up jets, which are mainly at low $$p_{\text {T}}$$, a jet vertex tagger [58], based on tracking and vertexing information, is applied for jets with $$p_{\text {T}}$$ $$< 60~\mathrm{GeV}$$  and $$|\eta | <2.4$$. Jets used in the event selection are required to have $$p_{\text {T}}$$ $$>30~\mathrm{GeV}$$ and $$|\eta | < 4.5$$. The $$\tau $$ leptons decaying to hadrons and $$\nu _{\tau }$$ are considered as jets as in previous analyses [16, 17].

The removal of overlapping candidate objects is performed in the following order. If any selected electron shares its ID track with a selected muon, the electron is removed and the muon is kept, in order to remove electron candidates originating from muon bremsstrahlung followed by photon conversion. If an electron lies a distance $\Delta R < 0.2$ of a candidate jet, the jet is removed from the event, while if an electron lies a distance $0.2< \Delta R < 0.4$ of a jet, the electron is removed. Muons lying a distance $\Delta R < 0.4$ with respect to the remaining candidate jets are removed, except if the number of tracks with $p_{\text {T}} > 0.5~\mathrm{GeV}$ associated with the jet is less than three. In the latter case, the muon is kept and the jet is discarded. Finally, if a jet lies a distance $\Delta R < 0.4$ of a candidate photon, the jet is removed.

The missing transverse momentum vector ${\varvec{E}}_{\text {T}}^{\text {miss}}$ is obtained from the negative vector sum of the momenta of the candidate physics objects, selected as described above. Calorimeter energy deposits and tracks are matched with candidate high-$p_{\text {T}}$ objects in a specific order: electrons with $p_{\text {T}}$ $> 7~\mathrm{GeV}$, photons with $E_{\mathrm {T}}^{\gamma }> 10~\mathrm{GeV}$, muons with $p_{\text {T}}$ $> 6~\mathrm{GeV}$ and jets with $p_{\text {T}}$ $> 20~\mathrm{GeV}$ [59]. Tracks from the primary vertex^3^ not associated with any such objects are also taken into account in the $E_{\text {T}}^{\text {miss}}$ reconstruction (“soft term”) [61].

Corrections are applied to the objects in the simulated samples to account for differences compared to data in object reconstruction, identification and isolation efficiencies for both the leptons and photons. For the photons, the efficiency corrections depend on whether or not they are converted, and on their $E_{\mathrm {T}}^{\gamma }$ and $\eta$; for the photons used in this analysis they are generally of the order of $1\%$.

## Event selection

The data were collected in *pp* collisions at $\sqrt{s}=13 ~\mathrm{TeV}$ during 2015 and 2016. The events for the analysis were recorded using a trigger requiring at least one photon candidate above a $E_{\mathrm {T}}^{\gamma }$ threshold of $140~\mathrm{GeV}$ to pass “loose” identification requirements, which are based on the shower shapes in the EM calorimeter as well as on the energy leaking into the hadronic calorimeter [62].

For events in the signal regions defined below, the efficiency of the trigger is more than 98.5%. The 1% difference in the efficiency between data and MC simulation is treated as a systematic uncertainty. Only data satisfying beam, detector and data-quality criteria are considered. The data used for the analysis correspond to an integrated luminosity of 36.1 fb$^{-1}$. The uncertainty in the integrated luminosity is ±3.2%. It is derived following a methodology similar to that detailed in Ref. [63], from a preliminary calibration of the luminosity scale using *x*–*y* beam-separation scans performed in August 2015 and May 2016.

Events are removed if they contain a bad-quality photon or jet [50, 64], arising from instrumental problems or non-collision background. Events are required to have a reconstructed primary vertex, as defined in Sect. 4.

Events in the signal regions (SRs) are required to have the leading photon satisfying the criteria defined in Sect. 4 and having $$E_{\mathrm {T}}^{\gamma }>150 ~\mathrm{GeV}$$, which is well above the thresholds used for the MC event generation and for the data-collection trigger. The |*z*| position, defined as the longitudinal separation between the beamspot position and the intersection of the extrapolated photon trajectory with the beam-line, is required to be smaller than 0.25 m. This criterion provides a powerful rejection against the muons from beam background [17], which can leave significant energy deposits in the calorimeters and hence lead to reconstructed fake photons that do not point back to the primary vertex. It is required that the photon and $${\varvec{E}}_{\text {T}}^{\text {miss}}$$ do not overlap in the azimuthal plane: $$\Delta \phi (\gamma ,~$$ $${\varvec{E}}_{\text {T}}^{\text {miss}}$$ $$) > 0.4$$. To further suppress the background events where the $$E_{\text {T}}^{\text {miss}} $$ is fake, e.g. arising from poorly reconstructed objects, a requirement on the ratio $$E_{\text {T}}^{\text {miss}}/\sqrt{\Sigma E_{\text {T}}} > 8.5~{\text {GeV}^{1/2}}$$ is added,^4^ where $$\Sigma E_{\text {T}} $$ is calculated as the scalar sum of all $$p_{\text {T}} $$ from the objects and the tracks contributing to the $$E_{\text {T}}^{\text {miss}} $$ reconstruction described in Sect. 4. This requirement mainly rejects the $$\gamma $$+jets background events. Events with more than one jet or with a jet with $$\Delta \phi (\mathrm {jets},~$$ $${\varvec{E}}_{\text {T}}^{\text {miss}}$$ $$)<0.4$$ are rejected (jet veto), the latter to remove events where $$E_{\text {T}}^{\text {miss}} $$ originates from jet mismeasurement. The remaining events with one jet are retained to increase the signal acceptance and reduce systematic uncertainties related to the modelling of initial-state radiation. Events are required to have no electrons or muons passing the requirements for $$e/\mu $$ candidates described in Sect. 4. This lepton veto mainly rejects *W* / *Z* events with charged leptons in the final state.

As the production of a pair of dark-matter candidates or of a high-mass BSM $Z(\rightarrow \nu \nu )\gamma$ resonance are both expected to lead to events with large $E_{\text {T}}^{\text {miss}}$, five SRs are defined with different $E_{\text {T}}^{\text {miss}}$ requirements: three inclusive (SRI1, SRI2 and SRI3) with increasing $E_{\text {T}}^{\text {miss}}$ thresholds and two exclusive (SRE1 and SRE2) with $E_{\text {T}}^{\text {miss}}$ in two different ranges. Table 2 shows the criteria for selecting events in the SRs and the number of events selected in data. The fraction of events in which the selected photon is unconverted is about $70\%$ for all regions. The fraction of selected events with no jets increases in the regions with lower $E_{\text {T}}^{\text {miss}}$ thresholds and ranges from about $50\%$ to about $70\%$.

**Table 2:** Criteria for selecting events in the SRs and the numbers of events selected in data

Event cleaning	Quality and primary vertex				
Leading photon	$$E_{\mathrm {T}}^{\gamma }> 150 ~\mathrm{GeV}$$, $$|\eta |<1.37$$ or $$1.52<|\eta |<2.37$$, tight, isolated, $$|z| < 0.25~{\text {m}}$$, $$\Delta \phi (\gamma ,~$$ $${\varvec{E}}_{\text {T}}^{\text {miss}}$$ $$) > 0.4$$				
$E_{\text {T}}^{\text {miss}}/\sqrt{\Sigma E_{\text {T}}}$	${>}8.5~{\text {GeV}^{1/2}}$				
Jets	0 or 1 with $p_{\text {T}} >30~\mathrm{GeV}$, $|\eta | < 4.5$ and $\Delta \phi (\mathrm {jets},~{\varvec{E}}_{\text {T}}^{\text {miss}})>0.4$				
Lepton	Veto on *e* and $\mu$				

	SRI1	SRI2	SRI3	SRE1	SRE2
$E_{\text {T}}^{\text {miss}} ~[{\text {GeV}}]$	${>}150$	${>}225$	${>}300$	150–225	225–300
Selected events in data	2400	729	236	1671	493
Events with 0 jets	1559	379	116	1180	263

## Background estimation

The SM background to the $\gamma +E_{\text {T}}^{\text {miss}}$ final state is due to events containing either a true photon or an object misidentified as a photon. The background with a true photon is dominated by the electroweak production of $Z(\rightarrow \nu \nu )\gamma$ events. Secondary contributions come from $W(\rightarrow \ell \nu )\gamma$ and $Z(\rightarrow \ell \ell )\gamma$ production with unidentified electrons, muons or with $\tau \rightarrow \text {hadrons}+\nu _{\tau }$ decays and from $\gamma$+jets events. The contribution from $t\bar{t}+\gamma$ is negligible thanks to the jet veto. The contribution from events where a lepton or a jet is misidentified as a photon arises mainly from *W* / *Z*+jets production, with smaller contributions from diboson, multi-jet and top-quark pair production.

All significant background estimates are extrapolated from non-overlapping data samples. A simultaneous fit in background-enriched control regions (CRs) is performed to obtain normalisation factors for the $W\gamma$, $Z\gamma$ and $\gamma$+jets backgrounds (see Sects. 6.1 and 6.2), which are then used to estimate backgrounds in the SRs; more details are given in Sects. 6.5.1 and 6.5.2. The same normalisation factor is used for both $Z(\rightarrow \nu \nu )\gamma$ and $Z(\rightarrow \ell \ell )\gamma$ in SR events. The backgrounds due to photons from the misidentification of electrons or jets in processes such as *W* / *Z*+jets, diboson and multi-jet events (referred to as fake photons) are estimated using data-driven techniques (see Sects. 6.3 and 6.4).

### $W\gamma$ and $Z\gamma$ backgrounds

For the estimation of the $W/Z\gamma$ background, three control regions are defined for each SR by selecting events with the same criteria used for the various SRs but inverting the lepton vetoes. As the $\gamma$+jets background contribution is not significant in these leptonic CRs, the requirement on the ratio $E_{\text {T}}^{\text {miss}}/\sqrt{\Sigma E_{\text {T}}}$ is not applied. In the one-muon control region (1muCR) the $W\gamma$ contribution is enhanced by requiring the presence of a muon; the 1muCR is sufficient to constrain the $W\gamma$ normalisation effectively without the need of a similar one-electron control region, which would be contaminated by $\gamma$+jets background. The two-lepton control regions enhance the $Z\gamma$ background by requiring the presence of a pair of muons (2muCR) or electrons (2eleCR). In each case, the CR lepton selection follows the same requirements as the SR lepton veto, with the addition that the leptons must be isolated with “loose” criteria [53] based on information from the calorimeter and tracking systems. In both 1muCR and 2muCR, the $E_{\text {T}}^{\text {miss}}$ value is computed disregarding muons, effectively treating them as non-interacting particles, in order to ensure that the $E_{\text {T}}^{\text {miss}}$ distributions in those CRs are similar to that in the SR. The same procedure is followed for electrons in 2eleCR. In both the $Z\gamma$-enriched control regions, the dilepton invariant mass $m_{\ell \ell }$ is required to be greater than $20 ~\mathrm{GeV}$, and the invariant mass of the leptons and photon, $m_{\ell \ell \gamma }$, is required to be smaller than $1~\mathrm{TeV}$ in order to avoid probing for potential BSM high-mass $Z\gamma$ resonances. The normalisation of the dominant $Z(\rightarrow \nu \nu )\gamma$ background source is largely constrained by the event yields in 2muCR and 2eleCR. The systematic uncertainty due to the extrapolation of the correction factors from CRs to SRs is taken into account (see Sect. 8).

### $\gamma$+jets background

The $\gamma$+jets background in the SRs consists of events where the jet is poorly reconstructed and partially lost, creating fake $E_{\text {T}}^{\text {miss}}$. This background, which increased in 2016 data with respect to 2015 data because of the higher pile-up conditions, is suppressed by the large $E_{\text {T}}^{\text {miss}}$ and jet–${\varvec{E}}_{\text {T}}^{\text {miss}}$ azimuthal separation requirements and by the requirement $E_{\text {T}}^{\text {miss}}/\sqrt{\Sigma E_{\text {T}}} > 8.5~{\text {GeV}^{1/2}}$ described in Sect. 5. This last requirement reduces the contribution of $\gamma$+jets events in SRI1 to less than 10% of the total background, with a negligible effect on the acceptance for signal events. The fraction of $\gamma$+jets events decreases with $E_{\text {T}}^{\text {miss}}$ and becomes less than $2\%$ of the total background in SRI3. For the estimation of the residual $\gamma$+jets background, a specific control region (PhJetCR) enriched in $\gamma$+jets events is defined. It uses the same criteria as used for the SRs, but does not apply the requirement on the ratio $E_{\text {T}}^{\text {miss}}/\sqrt{\Sigma E_{\text {T}}}$, and requires $85~\mathrm{GeV}< E_{\text {T}}^{\text {miss}} < 110~\mathrm{GeV}$ and azimuthal separation between the photon and ${\varvec{E}}_{\text {T}}^{\text {miss}}$, $\Delta \phi (\gamma ,~$ ${\varvec{E}}_{\text {T}}^{\text {miss}}$), to be smaller than 3.0. The latter selection minimises the contamination from signal events, which is estimated to be at most at the level of 1%. The PhJetCR is the same for all SRs; the systematic uncertainty due to the modelling of the $\gamma$+jets background, which affects its extrapolation from the low-$E_{\text {T}}^{\text {miss}}$ PhJetCR to the SRs with larger $E_{\text {T}}^{\text {miss}}$, is taken into account (see Sect. 8).

### Fake photons from misidentified electrons

Contributions from processes in which an electron is misidentified as a photon in the SRs are estimated by scaling yields from a sample of $e +E_{\text {T}}^{\text {miss}}$ events by an electron-to-photon misidentification factor. This factor is measured with mutually exclusive samples of $e^+e^-$ and $\gamma +e$ events in data. To establish a pure sample of electrons, the *ee* and the $e\gamma$ invariant masses ($m_{ee}$ and $m_{e\gamma }$) are both required to be consistent with the *Z* boson mass to within $10~\mathrm{GeV}$, and the $E_{\text {T}}^{\text {miss}}$ is required to be smaller than $40~\mathrm{GeV}$. Furthermore, the sidebands to the *Z* boson mass window are used to estimate and subtract possible contamination from misidentified jets in this sample. The misidentification factor, calculated as the ratio of the number of $\gamma +e$ to the number of $e^+e^-$ events, is parameterised as a function of $p_{\text {T}}$ and pseudorapidity and it varies between 0.59 and $2.5\%$. Systematic uncertainties in the misidentification factors are evaluated by varying the sideband definition, comparing the results of the method (with or without using the sideband subtraction) with generator-level information about $Z(\rightarrow ee)$ MC events, and comparing the misidentification factors in $Z(\rightarrow ee)$ and $W(\rightarrow e\nu )$ MC events. Background estimates are then also made for the four control regions, 1muCR, 2muCR, 2eleCR and PhJetCR, by applying the electron-to-photon misidentification factor to events selected with the same criteria used for these regions but requiring an electron instead of a photon. The estimated contribution from this background in the SRs and the associated uncertainty are reported in Sect. 7.

### Fake photons from misidentified jets

Background contributions from events in which a jet is misidentified as a photon are estimated using a sideband counting method [62]. This method relies on counting photon candidates in four regions of a two-dimensional space, defined by the isolation transverse energy and by the quality of the identification criteria. A signal region (region A) is defined by photon candidates that are isolated with tight identification. Three background regions are defined, consisting of photon candidates which are tight and non-isolated (region B), non-tight and isolated (region C) or non-tight and non-isolated (region D). The method relies on the assumption that the isolation profile in the non-tight region is the same as that of the background in the tight region. This has been verified in MC samples by checking that the correlation factor, calculated as ($N_{\text {A} }* N_{\text {D} }/ N_{\text {B}} * N_{\text {C}}$) is compatible with unity within uncertainties. The number of background candidates in the signal region ($N_{\text {A}}$) is calculated by taking the ratio of the two non-tight regions ($N_{\text {C}}/N_{\text {D}}$) multiplied by the number of candidates in the tight, non-isolated region ($N_{\text {B}}$). A correction to the method is added in order to take into account the leakage of real photon events to the three background regions. The systematic uncertainty of the method is evaluated by varying the criteria of tightness and isolation used to define the four regions. This estimate also accounts for the contribution from multi-jet events, which can mimic the $\gamma +E_{\text {T}}^{\text {miss}}$ signature if one jet is misreconstructed as a photon and one or more of the other jets are poorly reconstructed, resulting in large $E_{\text {T}}^{\text {miss}}$. This method is then used to evaluate the contribution of jets misidentified as photons in all analysis regions: the SRs and their associated four control regions, 1muCR, 2muCR, 2eleCR and PhJetCR. The estimated contribution from this background in the SRs and the associated uncertainty are reported in Sect. 7.

### Final background estimation

The normalisation factors for the $W\gamma$, $Z\gamma$ and $\gamma$+jets backgrounds are obtained via a profile likelihood fit (referred to as the background-only fit). For this fit, a likelihood function is built as the product of Poisson probability functions of the observed and expected event yields in the control regions. The event yield in the corresponding SR is not considered. The systematic uncertainties, described in Sect. 8, are treated as Gaussian-distributed nuisance parameters in the likelihood function. For each CR, the inputs to the fit are: the number of events observed in the data, the expected numbers of $W/Z\gamma$ and $\gamma$+jets background events, which are taken from MC simulations and whose normalisations are free parameters in the fit, and the number of fake-photon events obtained from the data-driven techniques.

Two different configurations are used for the fit: the background-only inclusive fit, which determines the normalisations for $W\gamma$, $Z\gamma$ and $\gamma$+jets backgrounds for each inclusive SR independently and the background-only multiple-bin fit, which determines the normalisations for the three exclusive SRs simultaneously. In the former case, four CRs corresponding to a given SR are used to obtain the normalisations. In the latter case, all ten CRs (the three leptonic CRs for each SR and the PhJetCR) associated with the three exclusive SRs are used. These fits are described in more detail in the following.

#### Background-only inclusive-SR fit

Background estimates in each inclusive SR are derived from a simultaneous fit to the respective four control regions (1muCR, 2muCR, 2eleCR and PhJetCR). The fitted values of the normalisation factors for $W/Z\gamma$ and $\gamma$+jets backgrounds (scale factors *k*) are reported in Table 3. Although the PhJetCR is defined in the same way for all SRs, the $k_{\gamma + {\mathrm {jets}}}$ factors in the three inclusive SRs differ slightly because they are fitted together with the other CRs, which are different for the different SRs.

The inclusive-SR fit is used to set model-independent limits, as shown in Sect. 9.

**Table 3:** Normalisation factors (scale factors *k*) obtained from a background-only inclusive-SR fit performed in each inclusive SR (the first three columns) and scale factors $k'$ obtained from a background-only multiple-bin fit performed simultaneously in the three regions SRE1, SRE2 and SRI3 (the last three columns), where $k'_{\gamma + {\mathrm {jets}}}$ applies to all exclusive signal regions. The errors shown include both the statistical and systematic uncertainties

Signal region	$E_{\text {T}}^{\text {miss}}$ [GeV]	$k_{W\gamma }$	$k_{Z\gamma }$	$k_{\gamma + {\mathrm {jets}}}$	$k'_{W\gamma }$	$k'_{Z\gamma }$	$k'_{\gamma + {\mathrm {jets}}}$
SRI1	${>}150$	$1.05\pm 0.09$	$1.10 \pm 0.09$	$1.07\pm 0.25$			
SRI2	${>}225$	$1.04\pm 0.11$	$1.14 \pm 0.13$	$1.06\pm 0.25$			
SRI3	${>}300$	$1.04\pm 0.15$	$1.27 \pm 0.23$	$1.06\pm 0.24$	$1.03\pm 0.14$	$1.27\pm 0.23$	
SRE1	150–225				$1.06\pm 0.10$	$1.10\pm 0.10$	$1.07\pm 0.25$
SRE2	225–300				$1.02\pm 0.12$	$1.09\pm 0.14$	

#### Background-only multiple-bin fit

A background-only multiple-bin fit is performed using simultaneously the control regions corresponding to the three signal regions SRE1, SRE2 and SRI3, which are mutually exclusive. The $\gamma$+jets normalisation factor is fixed in the common control region at low $E_{\text {T}}^{\text {miss}}$ (PhJetCR), while the $W\gamma$ and $Z\gamma$ normalisation factors are fitted in each $E_{\text {T}}^{\text {miss}}$ range separately. The estimated normalisation factors (scale factors $k'$) after this multiple-bin fit for each of the three SRs are also reported in Table 3. As expected, they agree within uncertainties with the scale factors *k* obtained from the inclusive-SR fit.

**Fig. 2:**
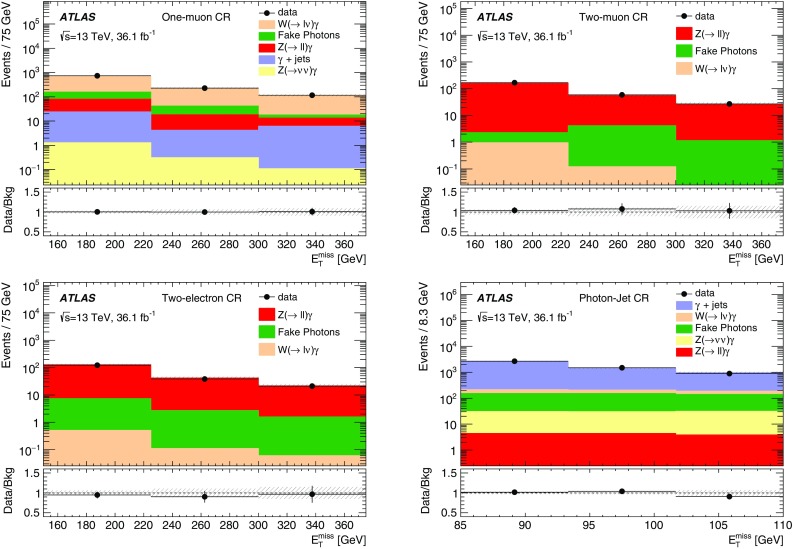
Distribution of $E_{\text {T}}^{\text {miss}}$ in data and for the expected total background in the CRs: 1muCR (*top left*), 2muCR (*top right*), 2eleCR (*bottom left*) and PhJetCR (*bottom right*). In 1muCR and 2muCR, the muons are treated as non-interacting particles in the $E_{\text {T}}^{\text {miss}}$ reconstruction; the electrons are handled similarly in 2eleCR. The total background expectation is normalised using the scale factors $k'$ derived from the multiple-bin fit. For 1muCR, 2muCR and 2eleCR, overflows are included in the third bin. The *error bars* are statistical, and the *dashed band* includes statistical and systematic uncertainties determined by the multiple-bin fit. The *lower panel* shows the ratio of data to expected background event yields

Post-fit distributions of $E_{\text {T}}^{\text {miss}}$ in the four control regions are shown in Fig. 2. The scale factors $k'$ from the multiple-bin fit are used for the different $E_{\text {T}}^{\text {miss}}$ ranges to produce these figures. These distributions illustrate the contribution from the different background processes.

The multiple-bin fit is used to set exclusion limits in the models studied, if no excess is found in the data, as discussed in Sect. 9.

## Results

Table 4 presents the observed number of events and the SM background predictions in SRI1 that is the most inclusive signal region with the lowest $E_{\text {T}}^{\text {miss}}$ threshold, as obtained from the simultaneous inclusive-SR fit to its CRs. The corresponding number of events is shown in the three lepton CRs and in PhJetCR. For the SM predictions both the statistical and systematic uncertainties, described in Sect. 8, are included.

Table 5 shows the observed number of events and the total SM background prediction after the fit in all signal regions. For SRI1, SRI2 and SRI3 regions the expected SM event yields are obtained from the inclusive-SR fit to each region; for SRE1 and SRE2 regions the expected SM event yields are obtained from the multiple-bin fit to the regions SRE1, SRE2 and SRI3. The expected SM event yields in SRI3 are the same when obtained from the multiple-bin fit. The numbers of observed events in the corresponding lepton CRs for each SR are also reported.

**Fig. 3:**
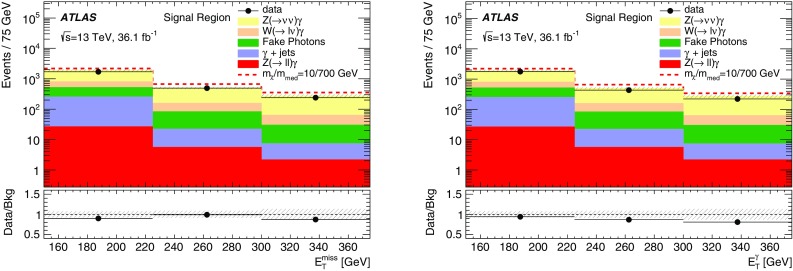
Distribution of $E_{\text {T}}^{\text {miss}}$ (*left*) and of $E_{\mathrm {T}}^{\gamma }$ (*right*) in the signal regions for data and for the expected total background; the total background expectation is normalised using the scale factors $k'$ derived from the multiple-bin fit. Overflows are included in the third bin. The *error bars* are statistical, and the *dashed band* includes statistical and systematic uncertainties determined by the fit. The expected yield of events from the simplified model with $m_{\chi } = 10~\mathrm{GeV}$ and an axial-vector mediator of mass $m_{\text {med}} = 700~\mathrm{GeV}$ with $g_{q}=0.25$ and $g_{\chi }=1.0$ is stacked on top of the background prediction. The *lower panel* shows the ratio of data to expected background event yields

The post-fit $E_{\text {T}}^{\text {miss}}$ and $E_{\mathrm {T}}^{\gamma }$ distributions are shown in Fig. 3 after applying the scale factors $k'$ from the multiple-bin fit. Only the $E_{\text {T}}^{\text {miss}}$ distribution is used in the multiple-bin fit, as discussed in Sect. 9.

**Table 4:** Observed event yields in 36.1 fb$^{-1}$ of data compared to expected yields from SM backgrounds in the signal region SRI1 and in its four control regions (CRs), as predicted from the simultaneous fit to CRs of SRI1 (see text). The MC yields before the fit are also shown. The uncertainty includes both the statistical and systematic uncertainties described in Sect. 8. The uncertainty on the pre-fit background is the pre-fit uncertainty, while the uncertainties on the fitted background are post-fit uncertainties. The latter are constrained by the fit as the use of control regions to normalise the dominant backgrounds allows to partially cancel some systematic uncertainties (see Sect. 8 for more details). The individual uncertainties can be correlated and do not necessarily add in quadrature to equal the total background uncertainty. The total fitted background does not match exactly the sum of the individual contributions because of the rounding applied

	SRI1	1muCR	2muCR	2eleCR	PhJetCR
Observed events	2400	1083	254	181	5064
Fitted background	$2600\pm 160$	$1083\pm 33$	$243\pm 13$	$193\pm 10$	$5064\pm 80$
$Z(\rightarrow \nu \nu )\gamma$	$1600\pm 110$	$1.7\pm 0.2$	–	–	$81\pm 6$
$W(\rightarrow \ell \nu )\gamma$	$390\pm 24$	$866\pm 40$	$1.1\pm 0.3$	$0.7\pm 0.1$	$163\pm 9$
$Z(\rightarrow \ell \ell )\gamma$	$35\pm 3$	$77\pm 5$	$233\pm 13$	$180\pm 10$	$13\pm 1$
$\gamma + {\mathrm {jets}}$	$248\pm 80$	$33\pm 8$	–	–	$4451\pm 80$
Fake photons from electrons	$199\pm 40$	$17\pm 3$	$0.50\pm 0.13$	$0.09\pm 0.04$	$72\pm 14$
Fake photons from jets	$152\pm 22$	$88\pm 19$	$7.9\pm 3.8$	$12\pm 5$	$284\pm 28$
Pre-fit background	$2400\pm 200$	$1025\pm 72$	$218\pm 15$	$181\pm 13$	$4800\pm 1000$

**Table 5:** Observed event yields in 36.1 fb$^{-1}$ of data compared to expected yields from SM backgrounds in all signal regions, as predicted from the simultaneous fit to their respective CRs (see text). The first three columns report the yields obtained from the inclusive-SR fit, while the two last columns report the yields obtained from the multiple-bin fit. The uncertainty includes both the statistical and systematic uncertainties described in Sect. 8. The uncertainties are post-fit uncertainties and are constrained by the fit as the use of control regions to normalise the dominant backgrounds allows to partially cancel some systematic uncertainties (see Sect. 8 for more details). The individual uncertainties can be correlated and do not necessarily add in quadrature to equal the total background uncertainty. The observed number of events in the four CRs relative to each SR is also shown. The total fitted background does not match exactly the sum of the individual contributions because of the rounding applied

	SRI1	SRI2	SRI3	SRE1	SRE2
Observed events	2400	729	236	1671	493
Fitted Background	$2600\pm 160$	$765\pm 59$	$273\pm 37$	$1900\pm 140$	$501\pm 44$
$Z(\rightarrow \nu \nu )\gamma$	$1600\pm 110$	$543\pm 54$	$210\pm 35$	$1078\pm 89$	$342\pm 41$
$W(\rightarrow \ell \nu )\gamma$	$390\pm 24$	$109\pm 9$	$33\pm 4$	$282\pm 22$	$75\pm 8$
$Z(\rightarrow \ell \ell )\gamma$	$35\pm 3$	$7.8\pm 0.8$	$2.2\pm 0.4$	$27\pm 3$	$5.7\pm 0.7$
$\gamma + {\mathrm {jets}}$	$248\pm 80$	$22\pm 7$	$5.2\pm 1.0$	$225\pm 80$	$17\pm 6$
Fake photons from electrons	$199\pm 40$	$47\pm 11$	$13\pm 3$	$152\pm 28$	$34\pm 8$
Fake photons from jets	$152\pm 22$	$37\pm 15$	$9.7^{+10}_{-9.7}$	$115\pm 24$	$27\pm 9$
Observed events in 1muCR	1083	343	116	740	227
Observed events in 2muCR	254	86	27	168	59
Observed events in 2eleCR	181	59	21	122	38
Observed events in PhJetCR	5064	5064	5064	5064	5064

## Systematic uncertainties

The systematic uncertainties are treated as Gaussian-distributed nuisance parameters in the likelihood function fitted to obtain the final background predictions in the SRs, as described in Sect. 6. The fit provides constraints on many sources of systematic uncertainty, as the normalisations of the dominant background processes are fitted parameters; only the uncertainties affecting the extrapolation between CRs and SRs therefore remain important.

The fitted uncertainties are presented as percentages of the total background predictions in SRs. The total background prediction uncertainty, including systematic and statistical contributions, varies from 6.1 to $14\%$ for the five SRs, dominated by the statistical uncertainty in the control regions, which varies from approximately 4.3 to $10\%$.

The relevant uncertainties (giving a contribution of more than 0.1% in at least one SR) are summarised in Table 6 for all SRs.

Aside from the uncertainty due to the statistical precision from the CRs, the largest relative systematic uncertainties are due to the uncertainty in the rate of fake photons from jets, which contributes 1.3–$5.3\%$, increasing for SRI3 and SRE2 because of the smaller number of events available for the estimation, and to the uncertainty in the jet energy scale, which contributes 1.4–$5.6\%$, decreasing in the regions with larger $E_{\text {T}}^{\text {miss}}$. The systematic uncertainty due to the modelling of the $\gamma$+jets background, which affects the extrapolation of this background from the PhJetCR to the SRs, is evaluated by independently varying the following four parameters with respect to the nominal values used in the MC generation: the renormalisation, factorisation and resummation scales by factors of 2.0 and 0.5, and the CKKW matching scale [65] to 15 and 30 GeV (the nominal value being 20 GeV). For the $W/Z\gamma$ backgrounds, the lepton identification/reconstruction efficiency uncertainties are propagated from the leptonic CRs to the SRs in terms of veto efficiency uncertainties. After the fit, the uncertainty in the luminosity [66] is found to have a negligible impact on the background estimation. The uncertainties due to the PDF have an impact on the $W/Z\gamma$ samples in each region but the effect on normalisation is largely absorbed in the fit, so their impact is negligible.

For the signal-related systematic uncertainties, the uncertainties due to PDF are evaluated following the PDF4LHC recommendations [67] and using a reweighting procedure implemented in the LHAPDF Tool [68], while uncertainties due to the scales are evaluated by varying the renormalisation and factorisation scales by factors of 2.0 and 0.5 with respect to the nominal values used in the MC generation. The uncertainties in initial- and final-state radiation, due to the choice of parton shower and multiple-parton-interaction parameters used with $\textsc {Pythia 8.186}$ are estimated by generating MC samples with the alternative tunes described in Ref. [31]. The PDF, scale and tune each induce uncertainties of up to about 5% in the acceptance (and cross section) in the simplified DM models.

**Table 6:** Breakdown of the relevant uncertainties in the background estimates for all SRs. The uncertainties are given relative to the expected total background yield after the inclusive-SR fit for SRI1, SRI2 and SRI3 and after the multiple-bin fit for SRE1 and SRE2. The total statistical uncertainty is dominated by the statistical uncertainty in CRs. The individual uncertainties can be correlated and do not necessarily add in quadrature to equal the total background uncertainty

	SRI1	SRI2	SRI3	SRE1	SRE2
Total background	2600	765	273	1900	501
Total (statistical+systematic) uncertainty	$6.1\%$	7.7%	14%	7.7%	8.8%
Statistical uncertainty only	$4.3\%$	6.2%	10%	5.5%	7.8%
Jet fake rate (Sect. 6.4)	$1.3\%$	$3.0\%$	$5.3\%$	1.7%	3.3%
Electron fake rate (Sect. 6.3)	$1.5\%$	$1.5\%$	$1.2\%$	1.5%	1.6%
Jet energy scale [56]	$4.1\%$	$1.9\%$	$1.4\%$	5.6%	0.6%
Jet energy resolution [69]	$0.7\%$	$0.2\%$	$0.5\%$	0.8%	0.3%
$E_{\text {T}}^{\text {miss}}$ soft-term scale and resolution [61]	$0.9\%$	$0.4\%$	$0.7\%$	1.1%	1.0%
Muon reconstruction/isolation efficiency [53]	$1.4\%$	$1.3\%$	$1.6\%$	1.3%	1.4%
Electron reco/identification/isolation efficiency [70]	$1.0\%$	$1.3\%$	$1.3\%$	0.8%	1.2%
Electron and photon energy scale [51]	$0.2\%$	$0.5\%$	$0.4\%$	${<}0.1\%$	${<}0.1\%$
Electron and photon energy resolution [51]	${<}0.1\%$	$0.3\%$	$0.2\%$	0.1%	1.0%
Photon efficiency [52]	$0.1\%$	$1.0\%$	${<}0.1\%$	0.2%	${<}0.1\%$
$\gamma +$jets modelling	$1.5\%$	$0.3\%$	$0.3\%$	2.3%	0.4%
$\langle \mu \rangle$ distribution in MC simulation (Sect. 3)	$1.3\%$	$0.3\%$	$0.9\%$	1.7%	0.3%

## Interpretation of results

The event yields observed in data are consistent within uncertainties with the predicted SM background event yields in all inclusive SRs, as shown in Table 5. The results from the SRs shown in Sect. 7 are therefore interpreted in terms of exclusion limits in models that would produce an excess of $\gamma +E_{\text {T}}^{\text {miss}}$ events. Upper bounds are calculated using a one-sided profile likelihood ratio and the $CL_S$ technique [71, 72], evaluated using the asymptotic approximation [73]. The likelihood fit includes both the SRs and their CRs.

The upper limits on the visible cross section, defined as the product of the cross section times the acceptance times the reconstruction efficiency defined in a fiducial region, $\sigma \times A \times \epsilon$, of a potential BSM signal, are obtained from the three inclusive SRs. The value of *A* for a particular model is computed by applying the same selection criteria as in the SR but at the particle level; in this computation ${\varvec{E}}_{\text {T}}^{\text {miss}}$ is given by the vector sum of the transverse momenta of all non-interacting particles. The *A* values with the selection for SRI1 or SRI2 or SRI3 are reported in Table 7 for the simplified DM models; the lowest values are found in models with low-mass off-shell mediators and the highest values in models with high-mass on-shell mediators. The observed and expected upper limits, at 95% confidence level (CL), on the fiducial cross section, defined as $\sigma \times A$ are shown in Table 7. They are computed by dividing the limit on the visible cross section by the fiducial reconstruction efficiency $\epsilon$ shown in the same table; as in the case of the acceptance, the efficiency is smaller in the high-$E_{\text {T}}^{\text {miss}}$ bins. The lowest efficiency for each signal region is used in a conservative way to set the fiducial cross-section limit. These limits can be extrapolated to models producing $\gamma +E_{\text {T}}^{\text {miss}}$ events once *A* is known, assuming that the conservative efficiency applies.

**Table 7:** The observed and expected limits at 95% CL on the fiducial cross section $\sigma \times A$. The values of the fiducial reconstruction efficiency ($\epsilon$), which is used for the calculation of the fiducial cross section, and of the acceptance (*A*) are also shown

Region	$\sigma \times A$ limit [fb]		
	SRI1	SRI2	SRI3
95% CL observed	7.0	3.7	2.3
95% CL expected	10.6	4.5	3.0
95% CL expected (${\pm }1\sigma$)	$14.5\pm 7.7$	$6.2\pm 3.3$	$4.2\pm 2.2$
*A* [%]	14–48	5–31	2–19
$\epsilon$ [%]	84–95	73–86	64–85

The expected limit on the signal strength in the simplified DM model is computed with the inclusive-SR fit for the various inclusive regions and with the multiple-bin fit in order to determine which strategy to adopt for limit setting. While SRI1 is the inclusive SR that gives the most stringent expected limits at very low DM/mediator masses, SRI2 is the inclusive SR providing the most stringent limits in the rest of the parameter space; SRI3, which has larger uncertainties, is not able to set better expected constraints on high-mass models in spite of their harder $E_{\text {T}}^{\text {miss}}$ spectra. The multiple-bin fit, making use of the expected signal distribution in $E_{\text {T}}^{\text {miss}}$ by combining the information from the various exclusive SRs, allows more stringent expected limits to be set than in any of the inclusive signal regions.

The results are presented for both the axial-vector and vector mediators using different couplings to show the complementarity to the direct searches in $X+E_{\text {T}}^{\text {miss}}$ events and the searches looking for the mediator, such as dijet or dilepton resonance searches, as recommended in Ref. [74]. Four models are considered with different mediators and different couplings to quarks, to DM particles, and to leptons, and these models are summarised in Table 8. As the choices of mediators and of couplings only affect the signal cross section and not the acceptance for signal events, the events generated for the axial-vector mediator with $g_q$ = 0.25, $g_{\chi }$ =1 and $g_{\ell } = 0$ (model A1), described in Sect. 3, can be re-scaled in order to obtain results for the other three models.

When placing limits in specific models, the signal-related systematic uncertainties estimated as described in Sect. 8 affecting $A \times \epsilon$ (PDF, scales, initial- and final-state radiation) are included in the statistical analysis, while the uncertainties affecting the cross section (PDF, scales) are indicated as bands around the observed limits and written as $\sigma _{\text {theo}}$.

**Fig. 4:**
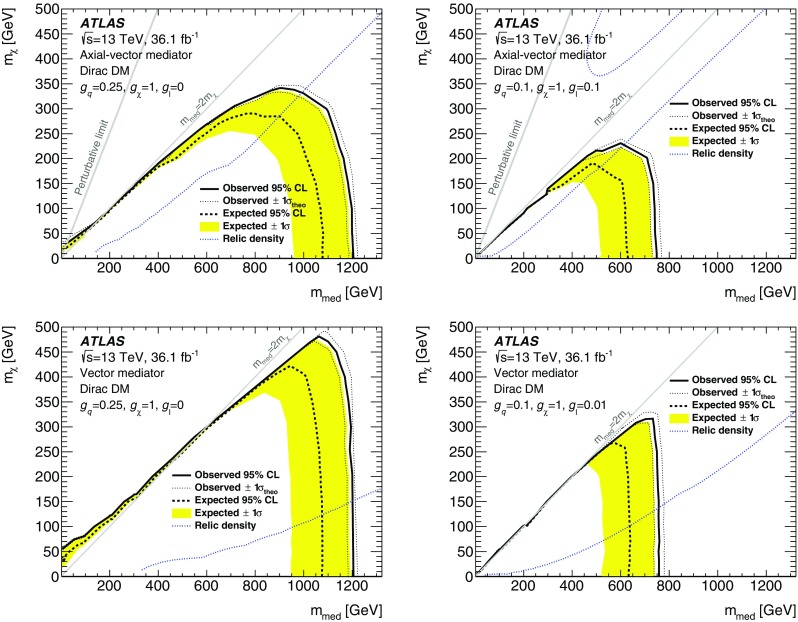
The observed and expected 95% CL exclusion contours for a simplified model of dark-matter production involving an axial-vector operator, Dirac DM and couplings $g_q = 0.25,$ $g_{\chi }=1$ and $g_\ell =0$ as a function of the dark-matter mass $m_{\chi }$ and the mediator mass $m_{\text {med}}$ (*upper left*). The plane under the limit curve is excluded. The same is shown for an axial-vector operator with couplings $g_q = 0.1, g_{\chi } = 1$ and $g_\ell = 0.1$ (*top right*), for a vector operator with couplings $g_q = 0.25, g_{\chi }=1$ and $g_\ell = 0$ (*bottom left*) and for a vector operator with couplings $g_q = 0.1, g_{\chi } = 1$ and $g_\ell = 0.01$ (*bottom right*). The region on the left is excluded by the perturbative limit which is relevant for axial-vector mediators [77]. The relic density curve [74, 76] is also shown: at higher mediator masses, the DM would be overabundant; at lower values, it would be underabundant; for the axial-vector scenario shown in the *upper right figure*, the region above the relic density curve at high dark-matter masses is also overabundant

Simplified models with explicit mediators are valid for all values of momentum transfer in *pp* collisions [10]. Figure 4 (top left) shows the observed and expected contours corresponding to a 95% CL exclusion as a function of $m_{\text {med}}$ and $m_{\chi }$ for the simplified model A1. The region of the plane under the limit curves is excluded. The region not allowed due to perturbative unitarity violation is to the left of the line defined by $m_{\chi } = \sqrt{\pi /2}m_{\text {med}}$ [75]. The line corresponding to the DM thermal relic abundance measured by the Planck collaboration [76] is also indicated in the figure; it is obtained as detailed in Ref. [74]. Figure 4 (top right) shows the contours for the A2 model, while Fig. 4 (bottom left) and (bottom right) show the contours for the V1 and V2 models, respectively. The search excludes mediator masses below the values reported in Table 8 for $\chi$ masses below the values reported in the same table. The limits for the model A1 are more stringent than the limits obtained with the 2015 data only [17], which excluded mediator masses below $710~\mathrm{GeV}$ for $\chi$ masses below $150~\mathrm{GeV}$.

**Table 8:** Observed limits at $95\%$ CL on the mediator mass and the DM particle mass for the four models considered. The mediators and couplings to quarks, to dark-matter particles and to leptons are specified for each model

Model	Mediator	$g_{q}$	$g_{\chi }$	$g_{\ell }$	Limit on $m_{\text {med}}$ [GeV] for low $m_{\chi }$	Limit on $m_{\chi }$ [GeV] reaching as high as
A1	Axial-vector	0.25	1	0	1200	340
A2	Axial-vector	0.1	1	0.1	750	230
V1	Vector	0.25	1	0	1200	480
V2	Vector	0.1	1	0.01	750	320

**Fig. 5:**
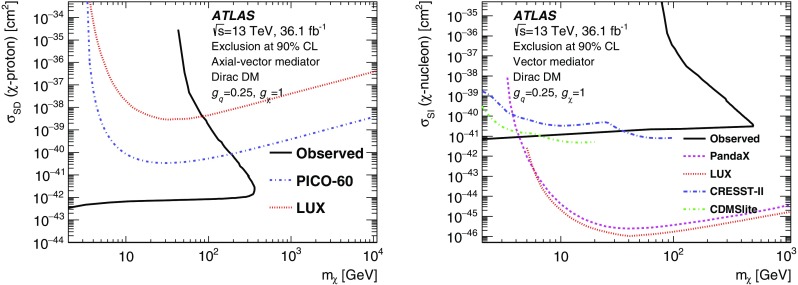
The 90% CL exclusion limit on the $\chi$–proton scattering cross section in a simplified model of dark-matter production involving an axial-vector operator, Dirac DM and couplings $g_q = 0.25,g_{\chi }=1$ and $g_\ell = 0$ as a function of the dark-matter mass $m_{\chi }$. Also shown are results at 90% CL from two direct dark-matter search experiments [78, 79] (*left*). The 90% CL exclusion limit on the $\chi$–nucleon scattering cross section in a simplified model of dark-matter production involving a vector operator, Dirac DM and couplings $g_q =0.25,g_{\chi } = 1$ and $g_\ell = 0$ as a function of the dark-matter mass $m_{\chi }$ (*right*); also shown are results at 90% CL from four direct dark-matter search experiments [80–83]

Figure 5 (left) shows the contours corresponding to a 90% CL exclusion translated into the plane of $\chi$–proton spin-dependent (SD) scattering cross sections vs. $m_\chi$ for the axial-vector mediator model A1. Bounds on the $\chi$–proton cross section are obtained following the procedure described in Ref. [77], assuming that the axial-vector mediator with couplings as in A1 is solely responsible for both collider $\chi$ pair production and for $\chi$–proton scattering. In this plane, a comparison with the result from direct DM searches [78, 79] is possible. The limit placed in this search extends to arbitrarily low values of $m_{\chi }$, as the acceptance at lower mass values is the same as the one at the lowest $m_{\chi }$ value shown here. The search provides stringent limits on the scattering cross section of the order of $10^{-42} {\text {cm}^2}$ up to $m_{\chi }$ masses of about $300~\mathrm{GeV}$. These results allow complementary limits to be set on the $\chi$–proton scattering cross section in the low DM mass region where the direct DM search experiments have less sensitivity due to the very low energy recoils that such low-mass dark-matter particles would induce. Figure 5 (right) shows the limit contours in the plane of the $\chi$–nucleon spin-independent (SI) scattering cross section vs. $m_{\chi }$ for the vector mediator model V1 compared with results of direct DM searches [80–83]. In this case the limit on the scattering cross section is of the order of $10^{-41} {\text {cm}^2}$ up to $m_{\chi }$ masses of about $500~\mathrm{GeV}$.

**Fig. 6:**
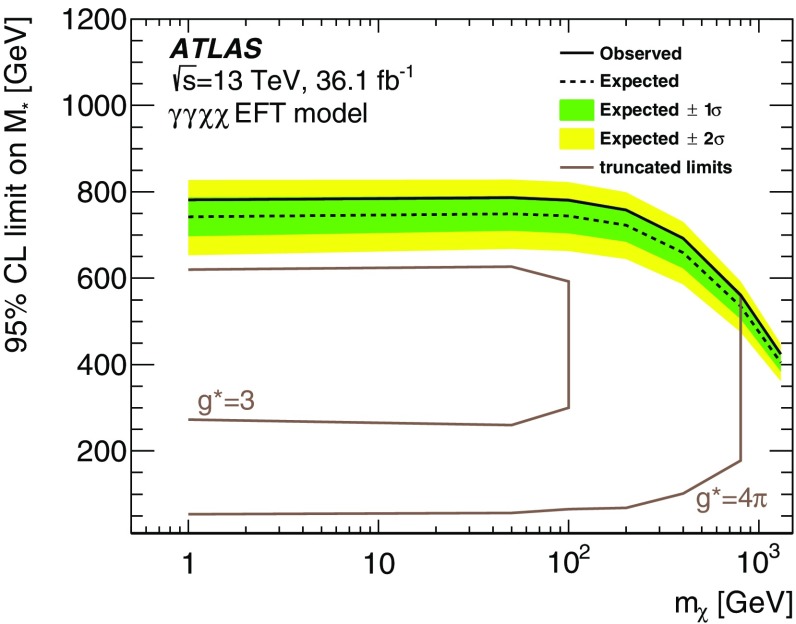
The observed and expected 95% CL limits on $M_{*}$ for a dimension-7 operator EFT model with a contact interaction of type $\gamma \gamma \chi \chi$ as a function of dark-matter mass $m_{\chi }$. Results where EFT truncation is applied are also shown, assuming representative coupling values, $g^*$, of 3 and 4$\pi$: for the maximal coupling value of 4$\pi$, the truncation has almost no effect; for lower coupling values, the exclusion limits are confined to a smaller area of the parameter space

In the case of the model of $\gamma \gamma \chi \bar{\chi }$ interactions, lower limits are placed on the effective mass scale $M_{*}$ as a function of $m_{\chi }$, as shown in Fig. 6. In this model, which presents a hard $E_{\text {T}}^{\text {miss}}$ spectrum, the signal events mainly contribute to the $E_{\text {T}}^{\text {miss}} >300~\mathrm{GeV}$ bin. The search excludes model values of $M_*$ up to about $790~\mathrm{GeV}$, which is a more stringent limit than the one placed in earlier searches [17]. The EFT description is not always valid at these scales. The effect of the truncation for two representative values of the EFT coupling, $g^{*}$, is shown in the same figure, assuming that the scale at which the EFT description becomes invalid ($M_{\text {cut}}$) is related to $M_{*}$ through $M_{\text {cut}}=g^{*}M_{*}$. For the maximal coupling value of 4$\pi$, the truncation has almost no effect; for lower coupling values, the exclusion limits are confined to a smaller area of the parameter space.

**Fig. 7:**
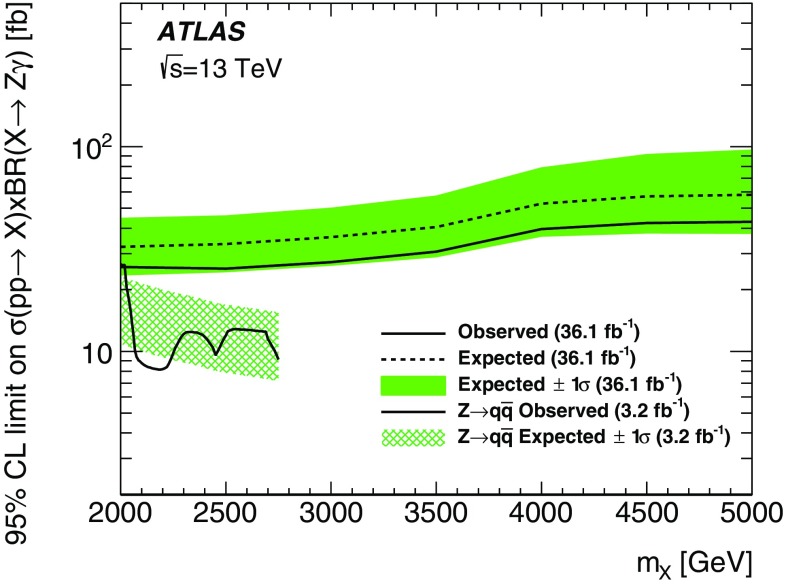
The observed (expected) limit at 95% CL on the production cross section of a $Z\gamma$ resonance as a function of its mass. The limits from the search in the $Z \rightarrow q\bar{q}$ channel with 3.2 fb$^{-1}$ [84] are also reported

The results are also interpreted in terms of a limit on the cross section for the production of a narrow heavy scalar $Z\gamma$ resonance produced through gluon–gluon fusion. Figure 7 shows the observed and expected limit at 95% CL on the production cross section of a $Z\gamma$ resonance as a function of its mass. The limit is produced in exactly the same way as the other signal samples, where an excess of events is sought in the three exclusive signal regions by using the multiple-bin fit. The heavy resonances are expected to populate mainly the $E_{\text {T}}^{\text {miss}} >300$  GeV signal region as they would have a hard $E_{\text {T}}^{\text {miss}}$ spectrum. The upper bound on $m_{\ell \ell \gamma }$ applied in 2eleCR and 2muCR (see Sect. 6.1) suppresses the contamination from potential high-mass $Z\gamma$ resonances in these control regions. Limits on such a resonance were also placed by bump searches in the very sensitive dileptonic channel and the hadronic channel for masses below and above 1.5 TeV, respectively [84]. Although the *Z* boson branching ratio to neutrinos is higher than to charged leptons, the presence of $E_{\text {T}}^{\text {miss}}$ makes the search in this channel much less sensitive than in the dileptonic channel; the region of interest for the analysis discussed here lies at higher masses, where it can complement the searches using *Z* boson hadronic decays whose limits, obtained with 3.2 fb$^{-1}$, are reported in the same figure. The observed (expected) limits at $95\%$ CL on the production of a $Z\gamma$ resonance are 26 and 43 fb (32 and 58 fb) for masses of 2 and $5~\mathrm{TeV}$, respectively.

## Conclusion

Results are reported from a search for dark matter in events with a high transverse energy photon and large missing transverse momentum in *pp* collisions at $\sqrt{s}=13~\mathrm{TeV}$ at the LHC. Data collected by the ATLAS experiment and corresponding to an integrated luminosity of 36.1 fb$^{-1}$ are used. The observed data are consistent with the Standard Model expectations. The observed (expected) upper limits on the fiducial cross section for the production of events with a photon and large missing transverse momentum are 7.0 and 2.3 fb (10.6 and 3.0 fb) at 95% CL for $E_{\text {T}}^{\text {miss}}$ thresholds of 150 and $300~\mathrm{GeV}$, respectively. For the simplified dark-matter model considered, the search excludes axial-vector and vector mediators with masses below 750–$1200~\mathrm{GeV}$ for $\chi$ masses below 230–$480~\mathrm{GeV}$ at 95% CL, depending on the couplings chosen. For an EFT $\gamma \gamma \chi \bar{\chi }$ model of dark-matter production, values of the suppression scale $M_*$ up to $790~\mathrm{GeV}$ are excluded at 95% CL and the effect of truncation for various coupling values is reported. The observed (expected) limits at 95% CL on the production cross section for a narrow $Z\gamma$ scalar resonance are 26 and 43 fb (32 and 58 fb) for masses of 2 and $5~\mathrm{TeV}$, respectively.

## Footnotes

1 The suppression scale, also referred to as $\Lambda$, is the effective mass scale of particles that are integrated out in an EFT. The non-renormalisable operators are suppressed by powers of 1/$M_{*}$.

2 ATLAS uses a right-handed coordinate system with its origin at the nominal interaction point (IP) in the centre of the detector and the *z*-axis along the beam pipe. The *x*-axis points from the IP to the centre of the LHC ring, and the *y*-axis points upward. Cylindrical coordinates ($r,\phi$) are used in the transverse plane, $\phi$ being the azimuthal angle around the beam pipe. The pseudorapidity is defined in terms of the polar $\theta$ angle as ${\eta = -\ln \left[ \tan (\theta /2)\right] }$.

3 The primary vertex is defined as the vertex with the highest sum of the squared transverse momenta of its associated tracks. It is reconstructed from at least two associated good-quality tracks with $p_{\text {T}} > 0.4~\mathrm{GeV}$ [60].

4 This ratio simulates the $E_{\text {T}}^{\text {miss}}$ significance because the $E_{\text {T}}^{\text {miss}}$ resolution due to purely calorimetric measurements scales approximately as $\sqrt{\Sigma E_{\text {T}}}$.
